# A peripheral blood-based approach involving vimentin along with AURKA enabled efficient tracking of elusive Oct4/Sox2-expressing disseminated breast cancer stem cells

**DOI:** 10.1042/BSR20253828

**Published:** 2026-06-24

**Authors:** Debomita Sengupta, Debanjan Thakur, Elizabeth Mahapatra, Salini Das, Jayanta Chakrabarti, Sagar Sen, Sutapa Mukherjee

**Affiliations:** 1 Department of Environmental Carcinogenesis and Toxicology, Chittaranjan National Cancer Institute, 37, S.P. Mukherjee Road, Kolkata 700026, India; 2 Department of Neurosurgery, The Houston Methodist Research Institute, Houston, Texas, U.S.A.; 3 Director & HoD, Department of Surgical Oncology, Chittaranjan National Cancer Institute, Hazra Campus: 37, S. P. Mukherjee Road, Kolkata-700 026, INDIA, Newtown Campus: Street No. 299, DJ Block, Action Area 1D, Kolkata – 700160, INDIA; 4 Department of Surgical Oncology, Chittaranjan National Cancer Institute, Street No. 299, DJ Block, Action Area 1D, Kolkata – 700160, INDIA.

**Keywords:** Atorvastatin, AURKA, Breast cancer stem cells, Oct4, Sox2, Vimentin

## Abstract

A major impediment in the therapeutic success of breast cancer (BC) arises from the persistence of clinically undetectable breast cancer stem cells (BCSCs) that need addressal by targeting translationally relevant markers to restrain relapse. Aurora kinase A (AURKA), due to its negative prognostic effect, was considered in clinical trials yet showed an unappreciable response, highlighting the need for additional markers. The present study tried exploring AURKA as a tool of relevance to detect the abundance of Oct4/Sox2(octamer-binding transcription factor 4/sex-determining region Y-box 2)-expressing BCSCs. The purpose was to understand the underlying intricacies governing the limited success of AURKA inhibition and to seek an improved relevant marker profile for detecting disseminated BCSCs. Flow cytometry and chromatin immunoprecipitation assay findings correlated Oct4/Sox2/AURKA expression, proposing an Oct4/Sox2 threshold-dependent AURKA induction. Surprisingly, in BC patient blood, Oct4/Sox2+ve cells were apparently lacking AURKA, emphasizing marker profile dynamicity in disseminating BCSCs with transient cell fate alterations. Immunoprecipitation/immunofluorescence results highlighted an interaction of pAURKA with fate-determinant pNUMB, hinting toward the existence of an AURKA/pNUMB axis for mesenchymal fate induction in breast cancer cells. Oct4/Sox2/AURKA+ve cells further expressed vimentin, highlighting a mesenchymal fate in BCSCs as evident from correlated Oct4/Sox2 and vimentin expression in patient blood. Hence, our study indicated an existing Oct4/Sox2/AURKA/pNUMB axis during transient mesenchymal differentiation of BCSCs and subsequently advocated for a peripheral blood-based approach using AURKA and vimentin for tracking BCSCs, thus supporting a co-targeting strategy. Preliminary *in vitro* intervention using combinatorial targeting of AURKA and vimentin reduced stemness propensities. Based on these interesting observations, further in-depth studies are warranted for clinical validation.

## Introduction

Breast cancer is the leading cause of cancer-related death in women globally, as per GLOBOCAN 2022 [1]. A large percentage of patients who demonstrated apparent clinical remission present with disease recurrence even after more than 10 years following initial diagnosis, which is indeed a matter of serious concern [2].

Disease recurrence happens to be primarily a consequence of the persistence of an apparently unidentifiable plastic population with chemo-refractory, radiation-resistant, and self-renewal/differentiation abilities [3–5]. These characteristic features are reminiscent of cancer stem cells (CSCs). With the discovery of these CSCs [6], multifarious approaches have been made to identify CSC-specific markers [7,8] to develop effective screening strategies, yet with limited success. The phenotypic switches selected by the stem cell niche contribute to a change of CSC marker profile, thereby failing the diagnostic protocols. Eventually, this population escapes the chemotherapeutic regimen due to their quiescent nature and chemotherapy being aimed at targeting the proliferating cells primarily, leading to metastasis and disease recurrence even after apparent clinical remission. Therefore, considering that the minor initial symptoms are frequently ignored by the patients, a cost-effective and simple detection system seems extremely necessary. Identification of the changing marker profile of breast cancer stem cells (BCSCs) during progression of the disease, therefore, appears inevitable.

Aurora kinase A (AURKA) seemed to hold potential as a prognostic marker in breast cancer with its reported gene amplification [9]. Enhanced transcriptional activity of AURKA by positive regulators like FOXM1, EGF [10], the TRAP200/MED1 complex, the β-catenin/TCF-4 complex [11], or Myc or inactivation of transcriptional repressors [12] of AURKA have also been documented, which presented AURKA as an attractive therapeutic target [13] and a probable diagnostic marker. AURKA is a serine threonine kinase responsible for spindle organization, assembly, and other mitotic functions [14]. In addition to its mitotic role, AURKA exhibits non-mitotic activities [15] like regulation of cell motility, senescence, and DNA repair capacities. AURKA is also involved in EMT [16] and BCSC self-renewal through induction of c-Myc, Sox2 (sex-determining region Y-box 2), and Nanog with enrichment of the CD44^hi^/CD24^low^ subpopulation [17]. Overexpression of AURKA in invasive breast cancer has already been reported [18] in 94% of cases, irrespective of histological subtypes. AURKA was proven to be a better prognostic marker than Ki67 in ER e breast cancer cases [19]. However, recently a phase III clinical trial using the selective AURKA inhibitor alisertib as a single agent reported no better prognosis in a 960-day time frame compared with the comparator arm consisting of gemcitabine/pralatrexate/romidepsin in relapsed or refractory peripheral T-cell lymphoma [20], indicating that AURKA expression alone failed to address the marker profile plasticity of disseminating CSCs.

In this context, the reported role of AURKA in cell fate determination in the breast appeared to be relevant [21]. Such profile plasticity in BCSCs may be expected to be attributed to AURKA itself and ultimately may lead toward a cell fate that shows undetectable AURKA expression. This admittedly demanded the inclusion of additional relevant markers that could address such AURKA-guided cell fate alterations. Considering the event of epithelial-mesenchymal transition (EMT), which is closely associated with AURKA as well as stemness [22], inclusion of EMT markers may provide the best benefit in addressing BCSC plasticity. Vimentin, a hallmark marker of EMT [23], has been associated with drug-resistant, aggressive features in breast cancer [24] with concomitant induction of stemness features [25] and increased relapses. Non-CSCs were reported to gain vimentin expression during conversion into CSCs [23]. Vimentin is, furthermore, associated with a stem-like signature in a model of mucoepidermoid carcinoma [26] and in a cervical cancer patient cohort following radiotherapy [27].

The present study thus sought to explore whether AURKA can be considered for the detection of disseminated BCSCs and to understand the possible mechanisms affecting its efficacy as a marker. Furthermore, the study aimed to develop a combinatorial approach with the inclusion of vimentin in the marker repertoire along with AURKA for efficient BCSC identification and subsequent targeting of the same.

## Materials and methods

### Gene expression analysis

For comparing AURKA transcript expression in tumor versus normal samples from TCGA data, the GEPIA2 platform was used. In the BRCA dataset, a separate box plot was generated across the histological subtypes with a Log_2_FC cutoff of 1, *P*-value cutoff of 0.01, and jitter size of 0.4.

### Survival analysis

For the calculation of relapse-free survival (RFS) in AURKA-high or AURKA-low breast cancer patients, Kaplan Meier Plotter for breast cancer has been used [28]. The dataset selected was 208079_s_at; patients were segregated using auto-select best cut-off, and JetSet best probe set was used as an option.

### Promoter analysis *in silico*

A DNA sequence up to 2 kb upstream of AURKA (Gene ID: 6790) transcription start site (TSS) was obtained from NCBI and used for analysis of transcription factor binding sites in ALGGEN-PROMO [29].

### Correlation analyses

Correlation of Oct4/Sox2 (octamer-binding transcription factor 4/sex-determining region Y-box 2) transcript expression with AURKA transcript expression was performed in GEPIA2 using the Pearson correlation coefficient, using a non-log scale for calculation and a log scale axis for visualization. The TCGA tumor as well as the normal dataset was used for Oct4/AURKA and the TCGA tumor, whereas the Genotype-Tissue Expression (GTex) normal datasets were used for Sox2/AURKA. Invasive breast carcinoma samples and paired breast samples from the TCGA database were considered for the analysis. In case the number of corresponding normal samples was not statistically fit to compare with the tumor samples, GTex normal sample data was taken into consideration. Correlation analysis between vimentin and the top 50 mesenchymal genes in breast cancer was performed using the microarray data from Alsuliman et al.’s EMT signature genes 2015–17 (breast cancer, PMID 26245467) as available in EMTome [30].

### Stemness signature matching and druggability assessment

Association of vimentin with stemness was assessed in StemChecker [31]. Stemness signature match and overlap in terms of percentage with stem cells were analyzed with Vim as an input query. Druggability assessment for vimentin was performed in SPIDER [32].

### Deriving drug indication associations for potential repurposing

Statins of either hydrophilic or lipophilic classes were assessed for their predicted approval likelihood in malignant neoplasms of the breast/malignant neoplasms with atorvastatin (lipophilic) and rosuvastatin (hydrophilic) as selected drugs. The present study was performed using a comprehensive web portal for discovering new drug association indications named RepurposeDrugs, available at https://repurposedrugs.org/ [33].

### Differential expression analysis and derivation of key enriched terms

A publicly available dataset, GSE206724 from GEO [34], was screened, and differentially expressed genes (*P*adj<0.05) were derived in Geo2r (control MCF-7 versus vimentin overexpressed). Pathway enrichment was performed in shinyGO [35] with significantly differentially expressed genes as input set at false discovery rate cutoff 0.05. The database used was KEGG (Kyoto Encyclopedia of Genes and Genomes) [36,37] and represented with the aid of the graphical tools.

### Cell lines, transfections, and treatments

Breast cancer cell lines MCF-7 and MDA-MB-231 were procured from the National Centre for Cell Science, Pune, India, and maintained in MEM (HiMedia, AT047—10× 1l) supplemented with 10% FBS (HiMedia, RM9970) with antibiotics, i.e., penicillin, streptomycin, and gentamycin, in a humidified CO_2_ incubator at 37°C. Prior to transfection, serum-containing media were replaced with Opti-MEM (Gibco, 11058-021) without antibiotics. pLKO.1 Sox2 3H b was a gift from Matthew Meyerson (Addgene plasmid #26352; http://n2t.net/addgene:26352; RRID:Addgene_26352); LL-hOCT4i-2 was a gift from George Daley (Addgene plasmid #12197; http://n2t.net/addgene:12197; RRID:Addgene_12197); and pLKO.1 puro was a gift from Bob Weinberg (Addgene plasmid #8453; http://n2t.net/addgene:8453; RRID:Addgene_8453). Plasmid DNA and Lipofectamine Stem Transfection Reagent (Invitrogen, 11668-030), diluted in Opti-MEM, was added to the cells for transfection purposes. Eventually, the efficiency of transfection was assessed in a BD LSRFortessa flow cytometer and analyzed using BD FACSDiva software.

MDA-MB-231 was exposed to a concentration of 50 nM alisertib (Selleckchem), dissolved in dimethyl sulphoxide (DMSO) (Merck D4540—100 ml), for 24 h. In another set of experiments, MDA-MB-231 cells were treated with 4 μM of atorvastatin calcium (Sigma–Aldrich, PHR1422-1G), dissolved in DMSO in accordance with previously published studies [38].

### Study approval

Surgically removed tissue samples, along with blood, were collected after signing the Informed Consent Form in accordance with the Helsinki Declaration. Blood was also collected from healthy women who participated voluntarily.

### Flow cytometry analysis

Breast cancer tissue samples were minced and treated with type IV collagenase (HiMedia, TC214—50 mg) to obtain a single-cell suspension. In case of blood, CTCs were isolated using Histopaque-1077 [39] and following the manufacturer’s (Sigma) procedure. Cell lines and mammospheres were trypsinized to obtain single cells. Eventually, the cells were fixed and permeabilized in chilled acetone for 20 min, washed in PBS, blocked, and treated with antibodies like Anti-Hu CD24 PE-Cyanine 7 (Invitrogen, 25-0247-42), Anti-Hu/Mo CD44 AlexaFluor (Invitrogen, 70056-0441-82), Aurora A Antibody (1F8) [Alexa Fluor® 647] (Novus Biologicals, NBP2-22118AF647), Aurora A Monoclonal Antibody (35C1) (Invitrogen, 45-8900), Vimentin antibody [VI-RE/1] (PE) (GeneTex, GTX79851), Vimentin Rabbit mAb (Abclonal, A19607), ABCG2 antibody (GeneTex, GTX100437), Oct-4 Antibody (Cell Signaling Technology, 2750), and Sox2 (D9B8N) Rabbit mAb (Cell Signaling Technology, 23064). Goat anti-rabbit IgG antibody, pre-adsorbed (FITC) (GeneTex, GTX03116), and goat anti-mouse IgG1 (heavy chain) antibody, F(ab′)_2_ fragment, pre-adsorbed (PE) (GeneTex, GTX04206-08), were used where applicable. All antibodies were used in dilutions recommended in the corresponding datasheets.

### Western blotting

Cells were lysed for western blot analysis following the standard laboratory protocol [40]. The antibodies used were Oct-4 Antibody (Cell Signaling Technology, 2750), Sox2 (D9B8N) Rabbit mAb (Cell Signaling Technology, 23064), Aurora A Monoclonal Antibody (35C1) (Invitrogen, 45-8900), and Vinculin (Abclonal, A2752) Rabbit mAb. Blots were developed with respective HRP-conjugated antibodies (Abclonal, AS014/AS003) and visualized with ECL substrate (Promega, W1015).

### Cell cycle

Cells were fixed in acetone [41], washed, and treated with RNase A (Sigma–Aldrich, R4875) at 37°C. The cells were then incubated in propidium iodide (Sigma–Aldrich, P4170) for 15 min, and flow cytometry analysis was performed.

### Chromatin immunoprecipitation

Cells were fixed by adding 1% formaldehyde (HiMedia, MB059—500 ml) to the media. Excess formaldehyde was quenched with glycine, and the media was aspirated off. Cells were subsequently lysed in lysis buffer containing protease inhibitor cocktail and sonicated further. Lysates were then precleared with Protein A/G plus agarose beads (Santa Cruz Biotechnology, sc-2003). A part of the lysate was stored in a −20°C freezer as input, and to the rest, antibodies, namely, Oct4 (Cell Signaling Technology, 2750S), Sox2 (Cell Signaling Technology, 23064S), and rabbit IgG isotype control (GeneTex, GTX35035), were added and kept in a rotator overnight at 4°C. The next day, the DNA–protein–antibody complexes were pulled down with beads, washed in low salt, high salt, and LiCl buffers, and eluted. Reverse crosslinking was performed at 65°C using 5M NaCl followed by proteinase K (SRL, 49936) treatment. Finally, DNA was purified by phenol-chloroform-isoamyl alcohol (SRL, 136112-00-0) extraction, resuspended in nuclease-free water (SRL, 96370), and processed for PCR. Sequences of the chromatin immunoprecipitation (ChIP) primers have been included in Table 1.

**Table 1:** Details of forward and reverse primer sequence for PCR following chromatin immunoprecipitation (ChIP) assay

Site	Forward primer sequence 5′–3′	Reverse primer sequence 5′–3′
1	GTGAGCACACGAGGACAAGA	GGTTGACAACAAACCCGACG
2	ACTTCTGCTGAGCACATCCC	CTTCCATTCCCACCCAGTCC
3	TGGGTTCAAGGAGGTCAGGA	GGGATGTGCTCAGCAGAAGT

### Indirect immunofluorescence

Cells were grown on sterile, poly l-lysine (Sigma, P8920) coated coverslips in cell culture plates. Cover slips, on the day of processing, were washed in chilled PBS and fixed in acetone for 10 min in −20°C freezer. The cover slips were then washed thrice in PBS and blocked with 1% FBS for 1 h at room temperature. After blocking, the cells were incubated overnight with AURKA antibody (Invitrogen, 45-8900) and pNUMB s276 antibody (Cell Signaling Technology, 9828S) as per the manufacturer’s recommended dilution and thereafter washed in PBS. Finally, the cells were stained in DAPI (Cell Signaling Technology, 4083S) and assessed under a fluorescence microscope (Leica) at 20× magnification or an Olympus laser scanning confocal microscope under 60× magnification.

### Co-immunoprecipitation

Protease inhibitors (Halt Protease Inhibitor, Thermo Scientific, 1862209) and antibodies (AURKA and pNUMB) as per the recommended dilution mentioned in the datasheet (2 μg/100–200 μg of protein) were added to tissue lysates and incubated at 4°C overnight. The IP suspensions were then transferred to a column (BioBasic, BS69020) containing a filter coated with Protein-A Sepharose beads and left at 4°C overnight. The next day, bead-antibody-protein conjugates were washed with 1× and 0.1× IP buffer as available in the kit. The purified protein complexes were then eluted with IP elution buffer and qualitatively analyzed for expression of the interacting protein candidates by western blotting.

### Mammosphere forming assay

Briefly, pretreated cells (vehicle/alisertib/atorvastatin/combination) were seeded onto ultralow attachment plates (Corning, 3261) in DMEM/F12 (Gibco, 11320-033) with added hEGF (Sigma, SRP3027—500 μg, 20 ng/ml), bFGF (Sigma, SRP3043—50 μg, 10 ng/ml), and 1× B27 (Gibco, 17504-044) and maintained for 7 days, after which they were imaged under an inverted microscope (Olympus, CKX41). Mammosphere-forming efficiency was calculated as (No. of mammospheres formed/No. of cells seeded) × 100%.

### Colony forming assay

An equal no. of cells (3 × 10^3^) was seeded following treatment and allowed to grow for 15 days. Formed colonies were fixed with methanol: acetic acid (3:1) and stained with 0.5% crystal violet (SRL, 074072). Colony-forming efficiency was calculated as (No. of colonies formed/No. of cells seeded) × 100%.

### Transwell migration assay

Cells were pretreated and seeded onto the upper chamber of 8 μm pore size Transwell inserts (Corning, 3464) in serum-free medium and allowed to migrate for 24 h. Migrated cells were fixed in 1% formaldehyde and stained with 0.1% crystal violet solution and counted under an inverted microscope (Olympus, CKX41).

### Statistical analysis

All the statistical analyses were performed using the GraphPad-Prism software (8.0.1). All the data have been acquired in replicates—as mentioned in the legends, placed in Prism for graphical representation and statistical analysis was performed for each graph on a case-to-case basis using a statistical method recommended by the software itself. The specific tests for calculating *P*-value and *R*-value have been mentioned in the concerned figure legends.

## Results

### AURKA expression is positively correlated with breast cancer relapse, probably driven by Oct4/Sox2

**Figure 1:**
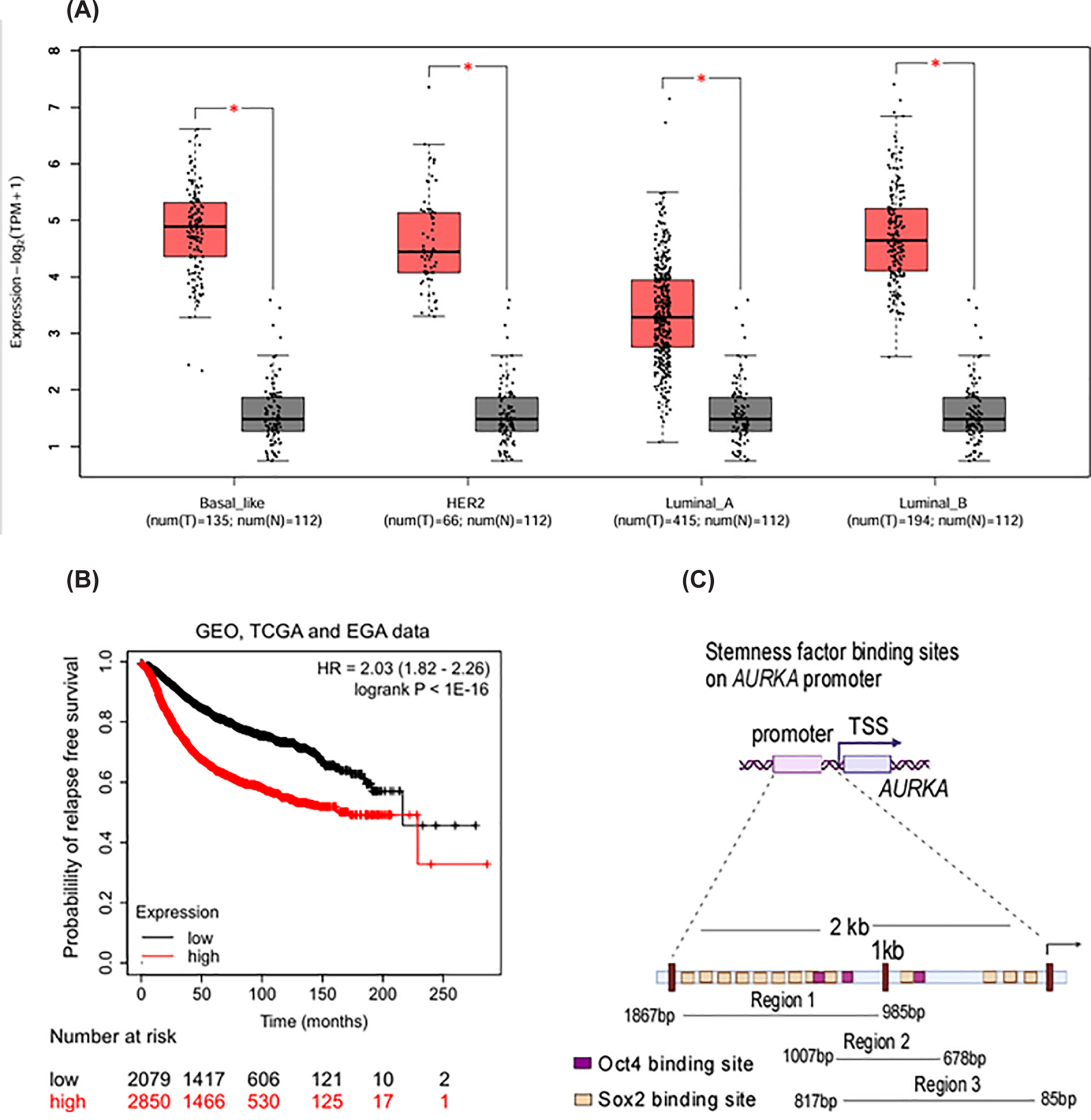
*AURKA* shows a negative correlation with relapse free survival of breast cancer patients (**A**) Box-whisker plots demonstrating overexpression of *AURKA* transcripts in tumor samples compared with adjacent normal samples as per the TCGA database. (**B**) KM-plotter analysis denoting a strong possibility of relapse among high-AURKA samples. (**C**) Diagrammatic representation demonstrating stemness factor binding sites within 2 kb upstream of *AURKA* TSS. Accordingly, Oct4 and Sox2 binding sites have been marked red and yellow, respectively.

To assess whether this enrichment of AURKA at the protein level in breast cancer cases is an outcome of increased transcript abundance, we performed a retrospective analysis of invasive breast carcinoma samples and paired breast samples from the TCGA database (Figure 1A). Results demonstrated significant up-regulation of the transcript in tumors compared with normal breast tissues across all the molecular subtypes, hinting towards deregulatory events occurring at the AURKA promoter of tumor samples. When the TCGA data was further screened, this high AURKA expression was found to be inversely proportional to the RFS of the patients (Figure 1B). With the notion that such reduced RFS may be a reflection of the interrelation between AURKA and cancer stemness, we screened the AURKA promoter up to 2kb upstream of the TSS for stemness factor binding sites. Our search unravelled several binding sites for Oct4 and Sox2 (Figure 1C). This observation suggested a predictable involvement of stemness factors in AURKA regulation. Based on these *in silico* findings, we wanted to gain a deeper insight into the involvement of Oct4/Sox2 in regulating AURKA and its subsequent clinical relevance in breast cancer.

### Oct4 and Sox2 are the major determining factors for influencing AURKA expression in breast cancer

**Figure 2:**
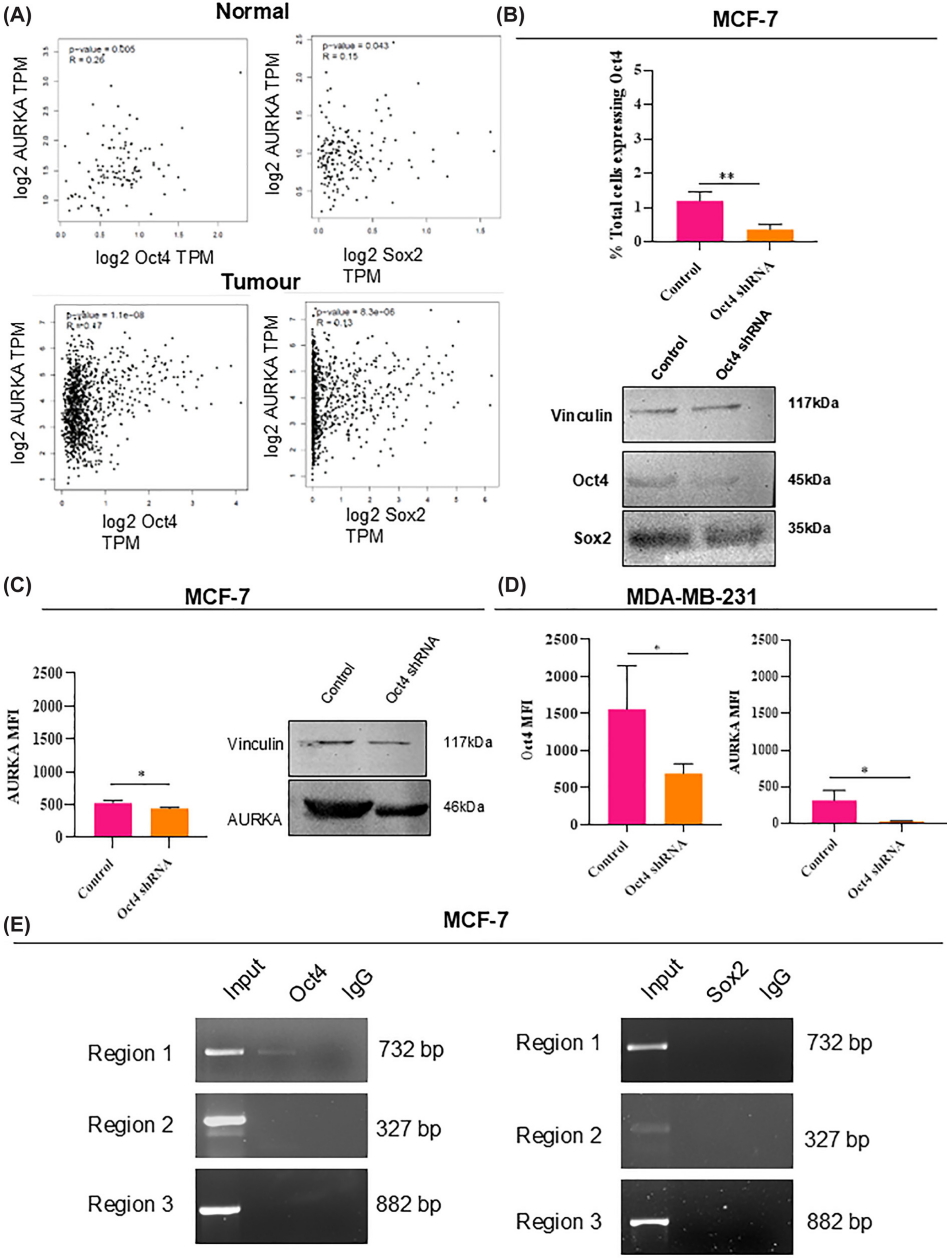
AURKA expression in breast cancer is primarily regulated by Oct4 (**A**) Scatter plots demonstrating positive correlation of *OCT4/SOX2* transcripts with *AURKA* transcripts as per TCGA data. (**B**) Interleaved bar diagrams representing confirmatory inhibition of Oct 4 as well as Sox2 in MCF-7 upon transfection with Oct4 shRNA, as observed by flow cytometric analysis and western blot. (**C**) Column graph (left panel) representing mean fluorescent intensities (MFIs) of AURKA-positive cells under vector control versus Oct4-shRNA transfection (right panel). (**D**) Column graph depicting diminished AURKA MFI subject to silencing of Oct4 in MDA-MB-231 cells. The experiments were performed in triplicates, and *P*-values were calculated using unpaired *t*-tests. *P<0.05, **P<0.01, ***P<0.001. (**E**) ChIP assay was performed in MCF-7 cells. The chromatin was immunoprecipitated by anti-Oct4 or anti-Sox2 antibody. Each precipitated DNA was analyzed by PCR, as evident in the corresponding gel images.

Based on the observations of Figure 1, correlation analysis between AURKA and Oct4 and Sox2 was carried out from TCGA data. Results indicated positive correlation of AURKA with both Oct4 and Sox2 at the transcript level (Figure 2A). In order to investigate whether such a correlation also prevails at the protein level, we made an attempt to silence Oct4 or Sox2 in MCF-7, a cell line that was previously reported to express both stemness factors [42]. Knockdown was confirmed by flow cytometry and western blot analysis. A gating strategy (Supplementary Figure S1) for the identification of Oct4/Sox2+ve cells has been shown. Confirmation of silencing of Oct4 (Figure 2B) and Sox2 (Supplementary Figure S2A) has been provided.

Thereafter, we checked AURKA expression by flow cytometry and western blot upon individual silencing. Interestingly, we observed that the expression of AURKA was diminished upon silencing either Oct4 (Figure 2C) or Sox2 (Supplementary Figure S2A). On the basis of the earlier evidential reports related to lower levels of Sox2 in MDA-MB-231 [42], we eventually attempted to overexpress Sox2 in order to understand its impact on AURKA expression in this cell line. We did observe elevation in AURKA expression upon Sox2 overexpression (Supplementary Figure S2B). Additionally, we reperformed silencing of Oct4 in MDA-MB-231 cells to understand its consequential impact, where reduced AURKA expression was apparent (Figure 2D).

We next explored whether such correlated expression was indeed an outcome of Oct4/Sox2-induced transcription of AURKA, subject to their recruitment at the upstream of AURKA TSS. For that purpose, we demarcated part of the genome spanning 2 kb upstream of the AURKA TSS into three different regions (regions 1, 2, and 3), and accordingly, forward and reverse primers were designed for three separate regions (Table 1). Subsequently, ChIP assays were conducted in the MCF-7 cell line, which authenticated the binding of Oct4, particularly in region 1, but not Sox2 (Figure 2E). This result emphasized regulation of AURKA by stemness factors, which, in the case of Oct4, was a result of direct recruitment upstream of TSS, indicating AURKA as a downstream target.

### Comparative expression profiles of AURKA and Oct4/Sox2 in breast cancer patients is reflective of their interdependence

The *in vitro* results supporting an interrelation between Oct4/Sox2 and AURKA prompted us to investigate the same in a patient setup. For that purpose, a total of 15 (*n* = 15) patients were recruited for the study. A summary table of patient samples has been tabulated in Table 2.

**Table 2:** Molecular subtype and therapy regimen details of recruited patients

Patient	Molecular subtype	Interventions	Age
1.	TNBC	NACT with first line doxorubicin/cyclophosphamide; docetaxel	45
2.	TNBC	No chemo	37
3.	ER+PR+HER2-	No chemo before surgery, follow up after 4 cycles of chemotherapy	35
4.	ER+PR+HER2-	NACT with first line doxorubicin/cyclophosphamide	51
5.	TNBC	No chemo	54
6.	ER-PR-HER2+	NACT 4 cycles; taxane+ 2 cycles trastuzumab	47
7.	ER+PR+HER2-	NACT with first line doxorubicin/cyclophosphamide	46
8.	DCIS	No chemo	63
9.	ER+PR+HER2+	NACT with first line doxorubicin/cyclophosphamide	44
10.	TNBC	NACT with first line doxorubicin/cyclophosphamide; docetaxel	37
11.	ER+PR-HER2-	No chemo	39
12.	ER+PR+HER2-	NACT with first line doxorubicin/cyclophosphamide	42
13.	ER+PR+HER2-	NACT with first line doxorubicin/cyclophosphamide	49
14.	ER+PR+HER2-	NACT 4 cycles	48
15.	ER+PR+HER2-	NACT with firstt line doxorubicin/cyclophosphamide; paclitaxel	52

**Figure 3:**
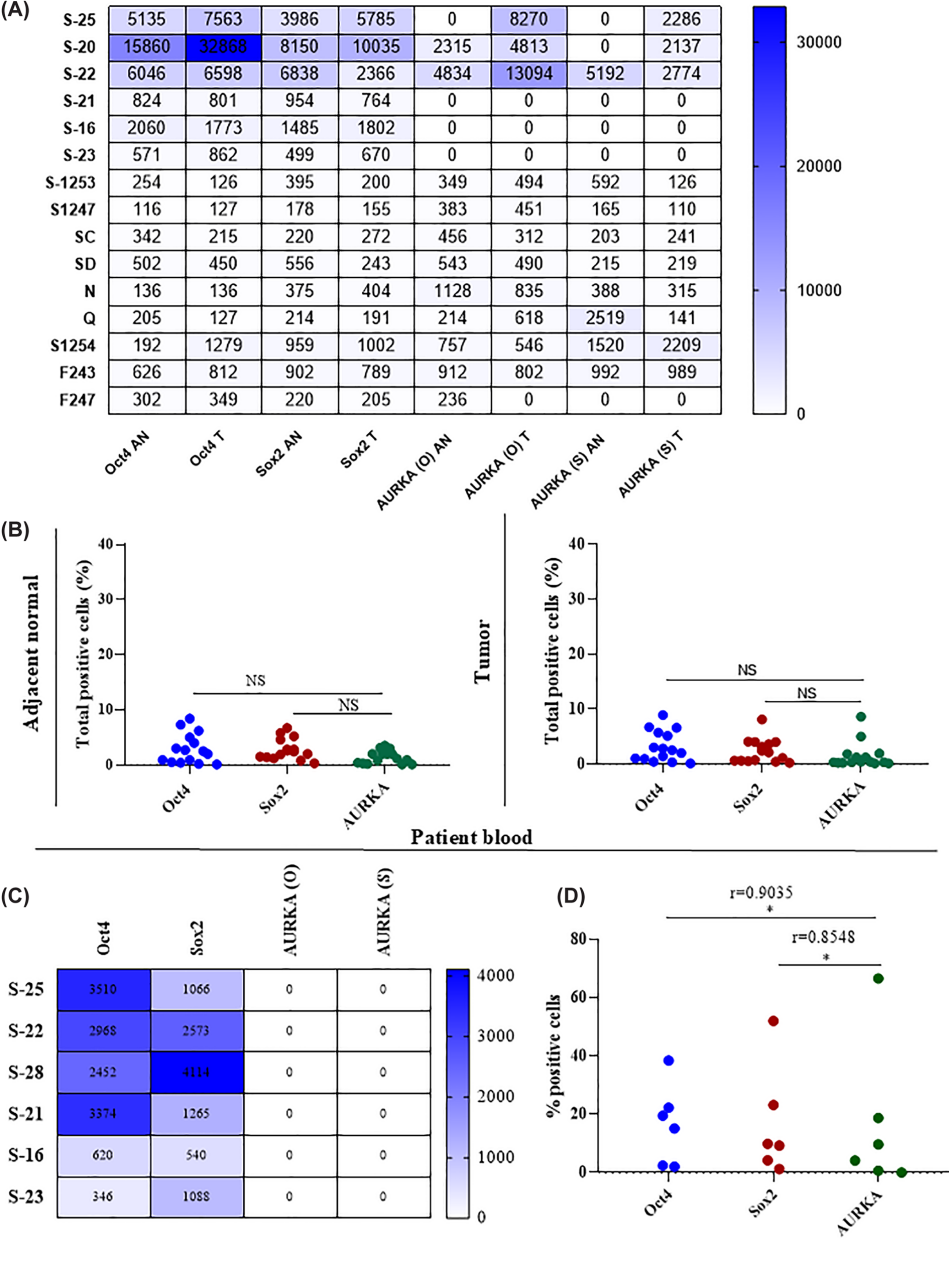
Expressional interdependence of AURKA and Oct4/Sox2 is evident in breast cancer patients (**A**) Heatmap representing Oct4/Sox2 MFI and AURKA MFI in Oct4 [AURKA(O)]/Sox2 [AURKA(S)] cells in adjacent normal samples (AN) and tumor (T) samples. (**B**) Scatter plots showing absence of correlation of total AURKA-positive cells with that of total Oct4/Sox2-positive cells in patient tissues; Left panel: adjacent normal and Right panel: tumor tissues. (**C**) Heatmap images representing the status of AURKA expression in Oct4/Sox2-positive cells in patient blood; the MFIs of Oct4/Sox2/AURKA have been included in the respective cells of the heatmap. (**D**) Scatter plots representing positive correlation of total AURKA-positive cells with total Oct4/Sox2-positive cells in patient blood. Significant correlations (*P<0.05) were derived by performing two-tailed tests and Pearson correlation analysis.

To explore this possibility, flow cytometry was performed. Considering the fact that normal tissues adjacent to breast tumors may undergo molecular alterations driven by ‘field cancerization’ [43] and thus affect disease prognosis, we got inclined toward a more holistic approach. Accordingly, all statistically relevant correlations were derived between Oct4/Sox2 and AURKA in both tumors and their corresponding adjacent normal breast tissues (Figure 3A). Interestingly, Oct4 expression (MFI) in the adjacent normal demonstrated a positive coherence with Sox2 expression in the adjacent normal (*r* = 0.932). Similar observations were also noted between Oct4 and Sox2 in corresponding tumors (*r* = 0.956). Oct4 expression in tumors was also further found to be positively correlated with Oct4 expression (*r* = 0.981) and Sox2 expression (*r* = 0.855) in adjacent normal tissue and Sox2 expression in tumors (*r* = 0.948). Having found this interrelation between Oct4 and Sox2, we next analyzed their correlation with AURKA. AURKA expression in Sox2-expressing cells of adjacent normal was positively correlated with AURKA expression in the Oct4-expressing cells of adjacent normal (*r* = 0.761) and tumor (*r* = 0.635), respectively. Similarly, AURKA expression in SOX2-expressing cells in tumor was correlated with AURKA expression in the Oct4-expressing cells of adjacent normal (*r* = 0.676) and tumor (*r* = 0.814).

AURKA expression in Oct4-expressing cells of adjacent normal is also correlated with Oct4 expression in the adjacent normal (*r* = 0.545) and tumor (*r* = 0.586). A similar relation was obtained for Sox2-expressing cells as well. All the corresponding *r*-values and *P-*values are tabulated in Supplementary Tables S1 and S2. Graphical representation of Oct4/Sox2 and AURKA expression in adjacent normal and tumor samples in terms of percentage is depicted in Figure 3B.

As shown in Figure 3C, AURKA was not detectable in circulating Oct4/Sox2-positive cells isolated from the peripheral blood of breast cancer patients, although the percent positivity of Oct4 and Sox2 were correlated with r values of 0.9035 between Oct4 and AURKA and 0.8548 between Sox2 and AURKA (Figure 3D).

All the correlation values and corresponding *P*-values were tabulated in Supplementary File S1

### Oct4/Sox2-induced transition toward mesenchymal fate is possibly driven by AURKA-NUMB interaction

**Figure 4:**
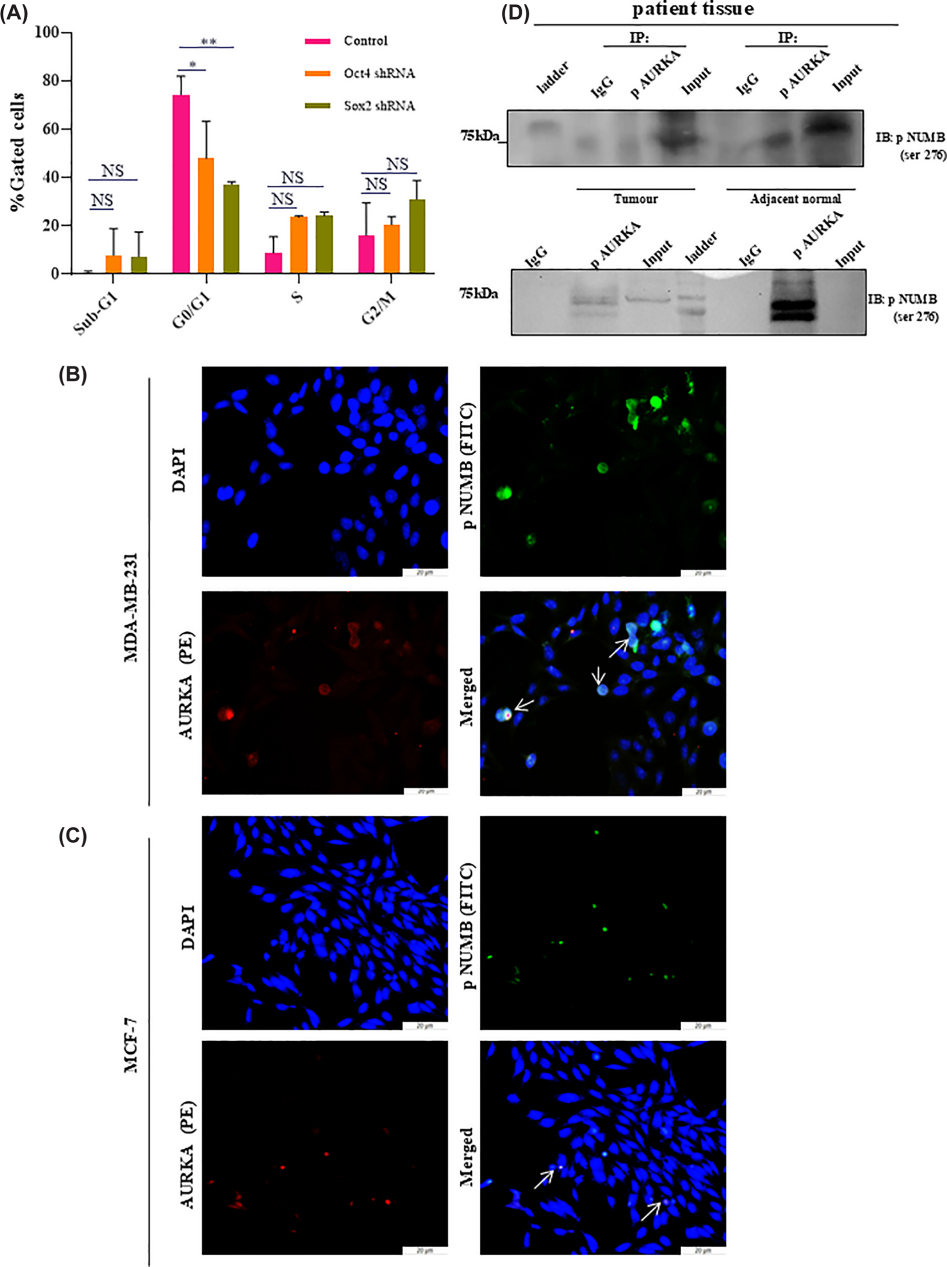
Oct4/Sox2 direct transition to mesenchymal fate through AURKA-NUMB interaction (**A**) Bar diagrams showing a decrease in G0/G1 population under Oct4/Sox2 silencing compared with vector control in MCF-7. Cell cycle assay was performed in replicates, and a two-way ANOVA followed by Tukey’s multiple comparisons test was used. *P<0.05, **P<0.01 were considered statistically significant. Representative immunofluorescence images demonstrating localization pattern of AURKA and phospho-NUMB(Ser-276) in MDA-MB-231 (**B**) and MCF-7 (**C**) cells. Magnification: 20×, Scale bar: 20 μm. (**D**) Immunoblots as obtained from Co-IP assay represented a physical interaction of phospho-AURKA with phospho-NUMB in breast tissue samples.

Undetectable AURKA expression in Oct4/Sox2-expressing cells in blood samples of breast cancer patients, as observed in Figure 3C, raised the possibility that such cells may be in a phase of the cell cycle characterized by diminished AURKA abundance. This possibility intrigued us to check the change in cell cycle distribution pattern *in vitr*o upon silencing either Oct4 or Sox2 separately, knowing from the earlier data that the knockdowns would lead to AURKA depletion in the same samples. Silencing of Oct4 or Sox2 impacted G0/G1 (Figure 4A) only, without any significant alterations in other cell cycle phases. This result indicated that Oct4 or Sox2 expression has some association with G0/G1 induction. Interestingly, G0/G1 has been linked with up-regulated expression of mesenchymal factors [44], upholding the probability that such differentiation may be mesenchymal. Gating strategies for checking transfection efficiency and histograms (Supplementary Figure S4) have been elaborated. The reported fate-determining function of AURKA in affecting mammary cell states [21] further guided us to check the localization status of an important cell-fate determinant (NUMB) in relation to AURKA. The result revealed colocalization of NUMB with AURKA during asymmetric as well as symmetric cell division, in an indirect immunofluorescence study (Figure 4B,C). This raised the question of how NUMB distinguishes between stem and non-stem-like daughter cells. Interestingly, a recent report aptly answered this problem, where they have reported that this asymmetry may be governed by numb phosphorylation status. We therefore explored the possibility of an interaction between AURKA and Numb in their phosphorylated forms. Co-immunoprecipitation (Co-IP) results confirmed a physical interaction between them in patient tissues, particularly enriched in the adjacent normal. Representative immunoblots of two different patient samples are shown (Figure 4D). Full-length western blot images were included (Supplementary Figure S5). This observation suggested the possibility of NUMB phosphorylation aided by AURKA, which may be involved in the attainment of an apparent mesenchymal state. Our findings were in alignment with earlier reports where Numb functionality loss by phosphorylation led to EMT. Moreover, it significantly proposes the involvement of the histologically normal tumor adjacent in the maintenance of the mesenchymal CSC pool. This is consistent with the concept of field cancerization and the reports regarding the value of tumor adjacency in affecting disease prognosis in breast cancer. These results thus indicated an Oct4/Sox2/AURKA-induced mesenchymal fate acquisition in breast cancer.

### The positive correlation between Oct4/Sox2 and vimentin in peripheral blood stems presumably from AURKA guided mesenchymal differentiation

**Figure 5:**
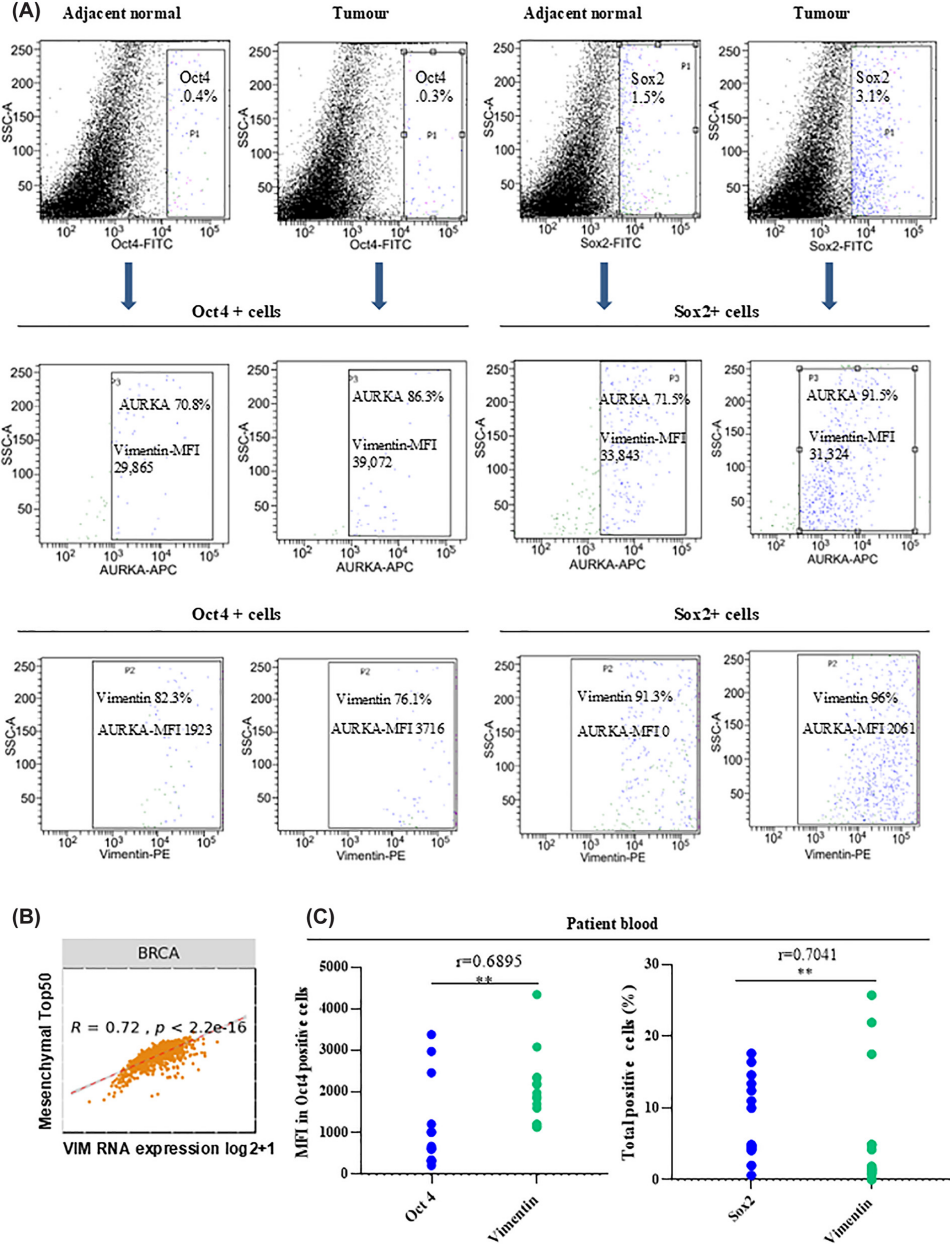
Vimentin showed a positive correlation with Oct4/Sox2 in peripheral blood (**A**) Flow cytometry scatter plots (upper panel) depicted Oct4 or Sox2 gated cells in tumor and corresponding adjacent normal of patient tissues. Middle panel representing MFI of vimentin in these AURKA-expressing Oct4- or Sox2-positive cells. Lower panel represents the MFI of AURKA in vimentin-expressing Oct4- or Sox2-positive cells. (**B**) Correlation plot demonstrating association of vimentin with mesenchymal gene signature in breast cancer (**C**) Scatter plots representing positive correlation of Oct4 (left panel) and Sox2 (right panel) with vimentin expression in patient blood. The values were statistically significant with *P<0.05. Correlations were derived by Pearson’s correlation analysis.

The findings so far indicated an association of AURKA with a mesenchymal differentiating phenotype. This was in coherence with previous reports, stating an association of AURKA with epithelial to mesenchymal transition [45] and loss of EMT-restraining activity of numb post-phosphorylation [46]. In light of these observations, we felt curious to explore whether AURKA-expressing cells acquire a fate oriented toward a transient mesenchymal state. We therefore analyzed the expression of the mesenchymal marker, vimentin, in Oct4/Sox2-expressing cells. Primary gating was done by either Oct4 or Sox2 (Figure 5A, upper panel), and they were gated further for AURKA. Both Oct4- and Sox2-expressing cells not only expressed AURKA but also were clearly showing pronounced expression of vimentin, as is noticeable from the high MFI (Figure 5A, middle panel). Augmented vimentin expression then compelled us to gate the Oct4- and Sox2-expressing cells with vimentin with the notion that such cells may represent the stem-like population with mesenchymal orientation. Vimentin expression was indeed evident in such cells, along with AURKA positivity for most. Significantly, we do also observe the presence of a vimentin-expressing Oct4/Sox2+ve population in the adjacent normal that did not show detectable AURKA expression (Figure 5A, lower panel). This may represent a population of stem-like cells with mesenchymal differentiation residing in the tumor periphery that would ultimately start tumor dissemination to blood. This was also partly observable from our results *in vitro*, which showed AURKA kinase inhibitor alisertib did decrease vimentin expression, yet in some populations of cells consistent expression of vimentin was still prevailing. Moreover, a population of cells also retained Oct4 expression following alisertib insult (Supplementary Figure S6). This may represent a set of cells that, even after alisertib challenge, remained unaffected owing to their already decided vimentin-expressing mesenchymal commitment.

To explore whether vimentin is actually a representative candidate of available mesenchymal gene signatures, we sought to find its association with the top 50 mesenchymal genes in EMTome web tool. Vimentin indeed demonstrated a positive correlation, further supporting our findings (Figure 5B). To further validate whether these Oct4/Sox2+ve blood cells with undetectable AURKA expression may have incurred a mesenchymal fate, we checked the association between Oct4 and Sox2 with vimentin in patient blood. Interestingly, a positive correlation (Figure 5C) was indeed found to be true for Oct4 and vimentin in terms of MFI (*r* = 0.6895) and for Sox2 and vimentin through percent positive cells (*r* = 0. 7041) despite their apparent AURKA negativity (Figure 3C). These results implied the presence of an apparently mesenchymally differentiated, vimentin-expressing Oct4+ve cell population in the patient’s blood that lost AURKA expression with a probable interdependency with circulating Sox2+ve cells as well. Additionally, the findings indicated toward an Oct4/Sox2/AURKA-induced modulation of numb functionality aiding vimentin-coupled mesenchymal transition.

### AURKA and vimentin are both to be considered as potential candidates for targeting drug-evading stem cell population in breast cancer

**Figure 6:**
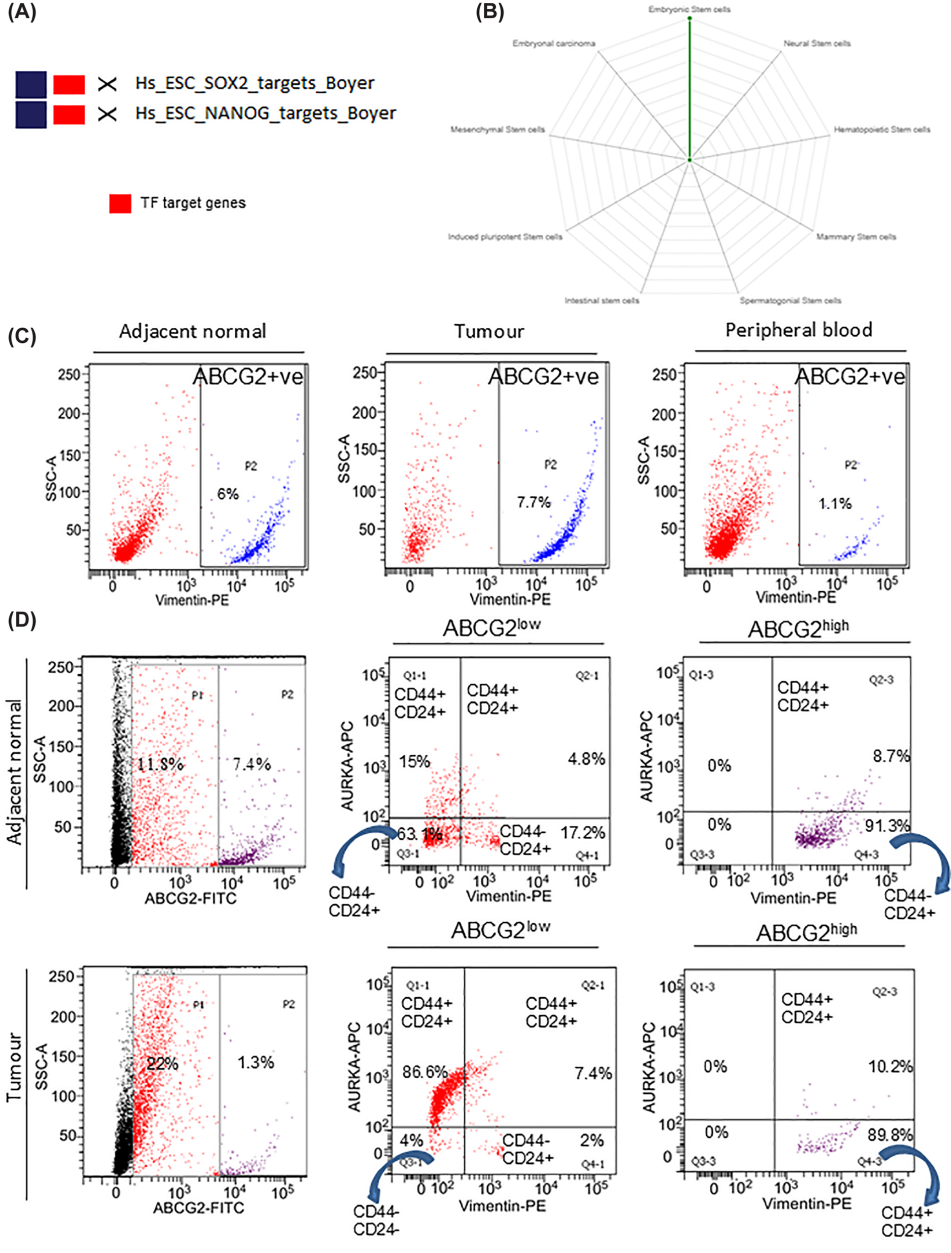
Both AURKA and vimentin emerged as dual targets for addressing drug-evading breast CSC population (**A**) Checkerboard representation displaying the inclusion of vimentin in stemness signatures available in StemChecker (**B**) Radar chart displaying the percent overlap of vimentin with stem cell types (**C**) Representative flow cytometry dot plots showing ABCG2 positivity in vimentin-expressing cells both in tissue and peripheral blood. (**D**) Flow cytometry dot plots in a representative tissue sample, gated for ABCG2. Upper panel denoted adjacent normal, and lower panel denoted tumor. ABCG2-low population was marked red, whereas ABCG2-high cells were marked violet.

Concerning the findings obtained so far displaying a positive correlation between vimentin and stemness factors, we felt curious to look for the existence of vimentin in available stemness signatures in the StemChecker web tool. We found a stemness signature profile match with vimentin, where vimentin was found to be a transcriptional target of stemness factors and also showed a stemness inclination towards embryonic stem cells, as depicted in Figure 6A,B. These *in silico* observations further supported our findings of an association of vimentin with stemness.

We therefore aimed to further validate whether vimentin-expressing cells in blood have truly disseminated from patient tissue upon therapy evasion; the expressional status of ABCG2, a drug resistance-associated CSC marker, was assessed in these vimentin+ve cells. The vimentin-expressing cells in the patient’s peripheral blood showed a matched phenotype of ABCG2 positivity with the corresponding patient tissue (Figure 6C, upper panel), indicating that they represent the drug-evading stem-like population with mesenchymal orientation and may be identified through vimentin tracking.

We further extended this study to look into the ABCG2-expressing population in patient tissue. Surprisingly, the flow cytometric dot plots (Figure 6D) revealed two distinct populations marked by low and high ABCG2. The ABCG2^low^ population showed a distinguishable increase in percentage in the tumor compared with adjacent normal (11.8% to 22%). We therefore felt curious to gate this ABCG2^low^ population with AURKA and vimentin. Additionally, their stemness profiles (CD44/CD24) were also examined. The AURKA+ve population in ABCG2^low^ gated cells showed a pronounced increase in tumor (in total, 19.8% in adjacent normal to 94% in tumor) with simultaneous double positivity towards CD44/CD24, indicating a retained stemness feature as evidenced in recent studies demonstrating increased stem-like capacities of such hybrid E/M cells [47]. This result implied that in tissues, the stem-like profile was still retained in the AURKA+ve cells, signifying the inevitable candidacy of AURKA for targeting stem-like cells at least in tissues. The ABCG2^high^ population, which was more abundant in the tumor adjacent, was predominantly vimentin^high^AURKA^low^, possibly due to initiation of mesenchymal differentiation as earlier observed in Figure 5. These cells were again observed to be mostly CD44/CD24 double-positive. Altogether, these findings are interesting in that they represented that the tumor core demonstrates features of both non-mesenchymal and stem-like mesenchymal cells, while the tumor adjacent seemed to be enriched in stem-like cells, directed toward mesenchymal fate as evident from their vimentin positivity. Moreover, flow cytometry results depicted enhanced vimentin positivity in Oct4/Sox2+ve cells in patient blood than in healthy volunteers (Supplementary Figure S7). These observations suggested that both AURKA and vimentin needs to be co-targeted to achieve the goal of minimizing disease progression in breast cancer.

### Vimentin emerged as a therapeutically addressable target owing to acquired deregulations of breast cancer-associated pathways upon overexpression

**Figure 7:**
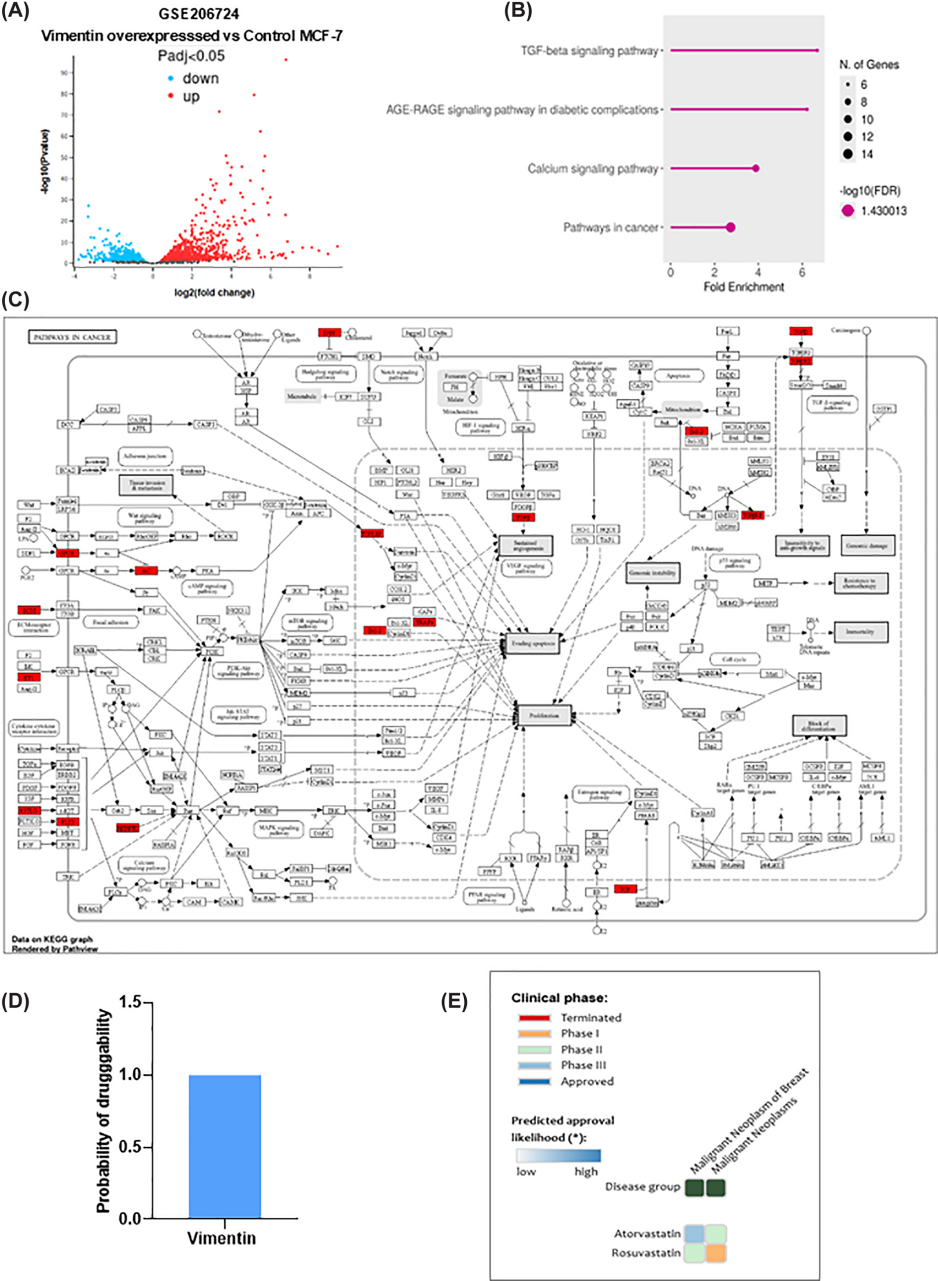
Induced deregulations of breast cancer associated pathways by vimentin (**A**) Volcano plot displaying differentially up-regulated or down-regulated genes induced by Vimentin. (**B**) Lollipop chart displaying the top enriched pathways derived from DEGs following vimentin overexpression. (**C**) KEGG pathway chart with vimentin induced DEGs were highlighted. (**D**) Column graph depicting the probability of druggability of vimentin. (**E**) Graphical representation of the predicted approval likelihood of atorvastatin in malignant neoplasm of breast.

The findings so far clearly denoted the importance of vimentin in addition to AURKA in addressing ABCG2-expressing cancer stem-like populations. These observations ushered in the need to probe for transcriptomic changes upon vimentin expression in a vimentin-null model of breast cancer. With this notion, available transcriptomic datasets were screened, and an altered transcriptomic profile upon vimentin overexpression could be discerned. This was evident from the volcano plot (Figure 7A), obtained from GEO2r, showing the differentially expressed genes (DEGs). For further derivation of key pathways that were altered due to vimentin expression, enrichment analysis was performed. The obtained lollipop chart represented pathways in cancer (fold enrichment 2.7), among others, as an enriched pathway pertaining to the DEGs (Figure 7B). A more detailed look at the pathways of cancer with the altered genes, demarcated in red, had been represented in the KEGG pathway chart (Figure 7C), highlighting the key cancer-associated pathways that may undergo a functional shift upon vimentin overexpression. These *in silico* analyses further strengthened our findings and steered us to consider vimentin as a marker of importance that may be opted for in targeting breast cancer. Druggability assessment was thus performed for vimentin, which also showed a high probability (0.998) as a drug target (Figure 7D). Considering published literature that demonstrated the potential of statins as possible drug candidates for breast malignancy through vimentin targeting [38], we further assessed the same (both hydrophilic and lipophilic statin groups) for repurposing in breast cancer, employing the RepurposeDrugs database. Heatmap derivations from the database displayed a predicted approval likelihood (Figure 7E), specifically for atorvastatin. These observations emphasized the candidacy of vimentin as a therapeutic target for breast cancer.

### Vimentin-expressing MDA-MB-231 cells displayed suppressed EMT and stemness features subject to administration of atorvastatin in combination with alisertib

**Figure 8:**
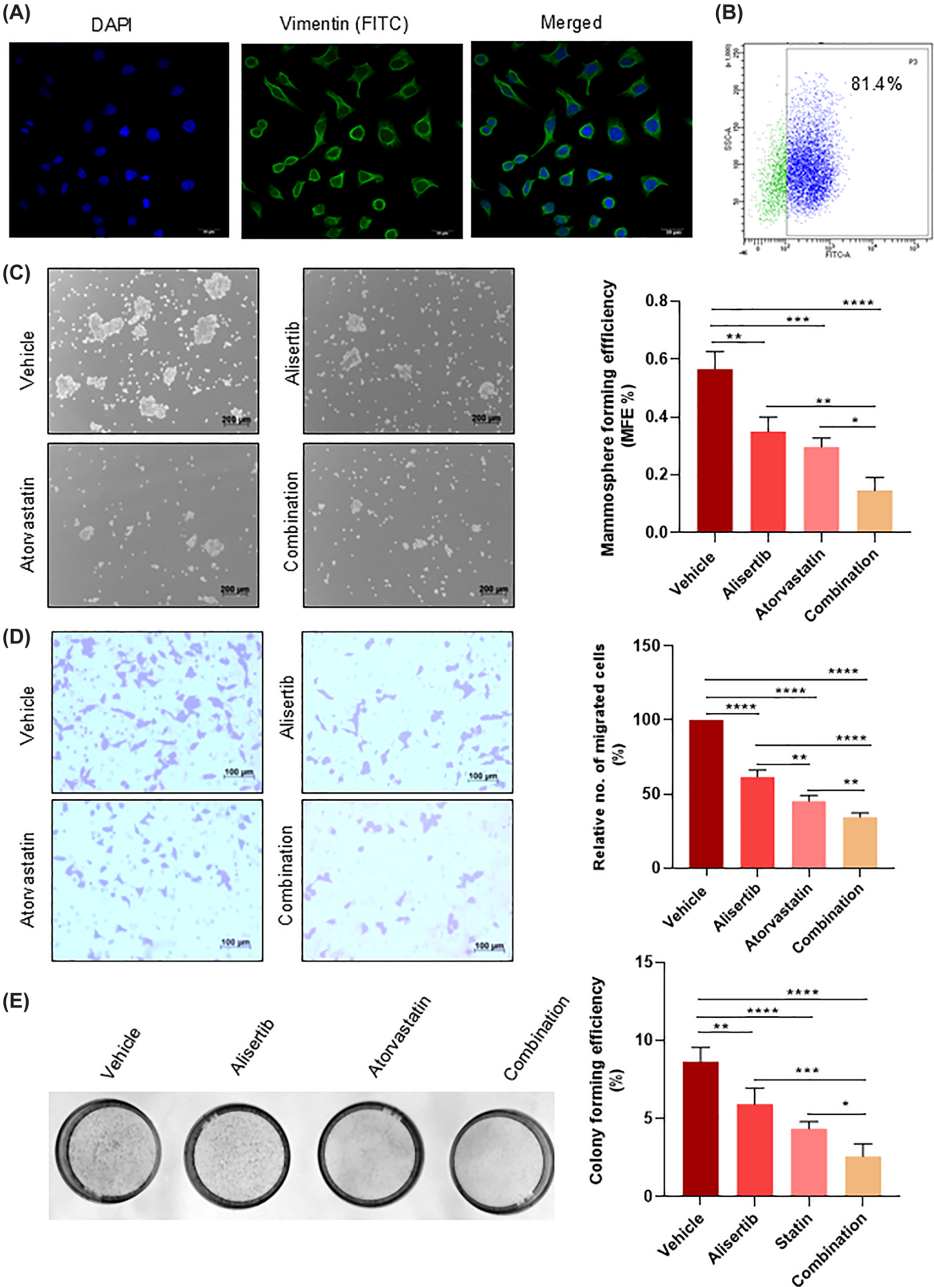
Atorvastatin in combination with alisertib suppressed EMT and stemness in vimentin-expressing MDA-MB-231 cells (**A**) Immunofluorescence images confirming vimentin (green) expression in MDA-MB-231 cells. Magnification: 60×, Scale bar: 20 μm. (**B**) Flow cytometry dot plots showing Vimentin positivity in MDA-MB-231 mammospheres. (**C**) Phase contrast images displaying altered mammosphere-forming ability in MDA-MB-231 under different treatment conditions. Changes in MFE% were represented graphically in the right panel. *P*<0.05 was considered statistically significant. Original magnification of the objective 4×. Scale bar: 200 μm. (**D**) Microscopic images of migrated MDA-MB-231 cells upon exposure to different treatment conditions. Original magnification of the objective 10×, Scale bar: 100 μm. Corresponding graph denotes change in no. of migrated cells relative to vehicle expressed as a percentage (right panel). (**E**) Representative images demonstrating changes in colony-forming efficacy upon exposure to different treatment conditions in MDA-MB-231. Right panel graphically illustrated the results in terms of changes in colony forming efficiency (%). Values were represented as mean ± SD from three independent experiments with *P<0.05, **P<0.01, ***P<0.001, ****P<0.0001. One-way ANOVA followed by Tukey’s multiple comparisons test as performed.

**Figure 9:**
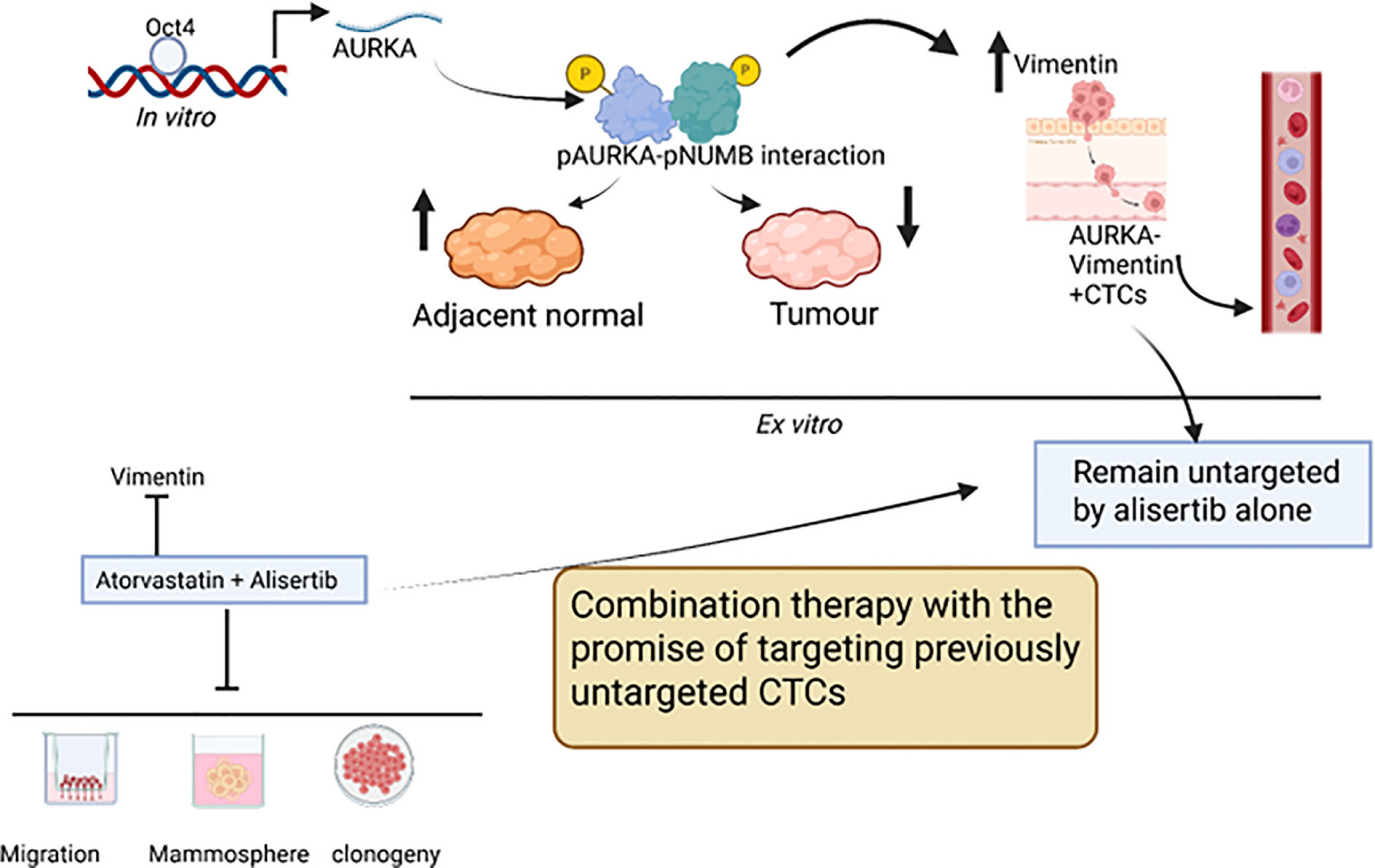
A representation of the overall study findings summarizing the improved efficacy of Atorvastatin–Alisertib combination in targeting BCSCs

Our observations so far indicated that although AURKA essentially participated in maintaining stem-like populations in tumors and normal adjacent but failed to express appreciably in circulating stem-like cells, which, however, expressed vimentin. Moreover, we observed a distinct influence of vimentin on cancer-associated pathways. These findings clearly evoked the need for a co-targeting strategy involving AURKA and vimentin to address both these populations. In consideration of a previous report showing the remarkable ability of atorvastatin to counteract vimentin expression in breast cancer and our *in silico* observations demonstrating the remarkable potential of atorvastatin in breast neoplasms, a combination of atorvastatin with AURKA inhibitor alisertib was strategized. To begin with, vimentin expression was checked and confirmed in MDA-MB-231 cells (Figure 8A). Hence, it was chosen for further studies. To assess the relevance of vimentin in breast cancer stemness, we enriched CSCs by mammosphere-forming assay and checked the expressional status of vimentin in the same. Our flow cytometry observations indeed found vimentin positivity in the formed mammospheres, which were in support of our *ex vivo* findings (Figure 8B). Having found vimentin positivity in mammospheres, we next aimed to observe the impact of atorvastatin on vimentin expression. The flow cytometry result showed reduction in the percentage of vimentin-expressing cells (Supplementary Figure S8). Thereafter, mammospheres were treated with either atorvastatin alone or in combination with alisertib for mammosphere-forming ability. Cells treated with a combination regimen displayed the lowest mammosphere-forming ability, as evident from the MFE% (Figure 8C). Combination treatment not only lowered mammosphere formation but also curbed the migratory capacity of MDA-MB-231 (Figure 8D), possibly indicative of the efficacy of the combinatorial regimen in restricting EMT. Moreover, alisertib and atorvastatin combination also fairly diminished clonogenic expansion as observed from the reduced colony-forming efficiency (Figure 8E) in the clonogenic viability assay. All these findings thus propose a combination targeting strategy against vimentin-expressing CSCs in breast cancer. The overall experimental findings have been schematically depicted (Figure 9).

## Discussion

Our *in silico* studies initially observed elevated AURKA abundance in breast tumors, which further negatively influenced RFS. This observation guided our study in finding out the probable role of stemness factors in the regulation of AURKA, considering the possibility that AURKA abundance may reflect the abundance of stem-like cells. Our *in silico* and *in vitro* studies, along with correlative expression of Oct4/Sox2 and AURKA as identified in patient tissues and blood, either in the same cells (tissue) or in total (total percentage), indicated AURKA to be regulated by stemness factors. This observation was indeed an outcome of direct transcriptional regulation of AURKA through direct promoter binding of Oct4. However, for Sox2, rather than representing direct binding to the AURKA promoter, the observed effect may be a consequence of the close functional interrelationship between Oct4 and Sox2 as previously reported [48], since Oct4 knockdown resulted in down-regulation of Sox2 as well. Thus, regulation of AURKA by Sox2 appears to be functionally inferred rather than directly demonstrated.

However, Oct4/Sox2+ve cells in blood did not display detectable AURKA expression. These findings led us to hypothesize that tissue-resident Oct4/Sox2/AURKA+ve breast cancer cells might have undergone a fate transition to a state that is characterized by undetectable AURKA expression. Oct4/Sox2, the regulators of AURKA, contributed in maintaining the cells in G0/G1 phase *in vitro*. This may partly explain the apparent AURKA negativity of Oct4/Sox2-expressing cells in blood, as it is reported that the most pronounced expression of AURKA occurs during G2/M phase [49]. Another interesting report [50] demonstrated that fluctuating levels of Oct4/Sox2 impacted cell fate commitment, primarily in G1 but not in other phases of cell cycle. This report compelled us to think of an Oct4/Sox2-mediated transition of cell fate upon G0/G1 induction. This may involve AURKA, considering its role in determining mammary cell fate as documented previously [21], where Notch-dependent function of AURKA in mitotic spindle orientation was evidenced. Interestingly, in addition to AURKA, NUMB also regulates cell fate decisions through Notch. NUMB additionally restricts EMT [51] and curbs the expansion of stem cell reservoir, a functionality that is lost upon phosphorylation [46]. Significantly, our findings revealed a more pronounced interaction between pNUMB/pAURKA in the adjacent normal, supposedly directing towards the loss of restraining capacity of NUMB by AURKA during the event of EMT. The association between G0/G1 as induced by Oct4/Sox2 and EMT factors [44] coupled with the reported role of AURKA in EMT [52] further strengthened our hypothesis. Our eventual findings indeed observed mesenchymal transition as demonstrated by the elevated vimentin expression of Oct4/Sox2+ve AURKA-expressing cells. Furthermore, a positive correlation between vimentin and Oct4/Sox2 in patient blood also reflected the same. Dissemination of BCSCs in blood was reported to be preceded by the formation of hybrid mesenchymal BCSC populations through engulfment of mesenchymal stem or stromal cells by breast cancer cells [53]. Our results were in agreement with the mentioned report regarding the dissemination of BCSC-like mesenchymal cells and thus strengthened the evidence of involvement of the stemness factors Oct4/Sox2 in mesenchymal cell-fate commitment and eventual dissemination. The observations from this study further emphasized the inclusion of AURKA in addition to Oct4/Sox2 in determining the mentioned cell fate. Vimentin positivity was also notable in Oct4/Sox2-positive cells in addition to AURKA. However, in adjacent normal, interestingly, we noticed a population that lacked detectable AURKA but showed vimentin expression. Moreover, we detected a persistent population in MDA-MB-231 that retained vimentin expression even after alisertib-mediated inhibition of kinase activity of AURKA. Although it is difficult to interpret in the *in vitro* context, still considering that cell lines themselves still maintain discernible heterogeneity, it might be speculated that these cells had already lost their dependence on AURKA to maintain vimentin-expressing mesenchymal state. These findings were in further coherence with our *ex vivo* observations of a persistent population without detectable AURKA but enriched vimentin expression. Moreover, we also observed the presence of a cell population with retained Oct4 expression even after alisertib insult, further raising the possibility of maintenance of a refractory population even after AURKA targeting.

Whether the tumor periphery harboring the apparently ‘normal’ tissue serves as the major site where the mesenchymal hybrid stem cells are generated and eventually disseminated is yet to be confirmed in a larger patient cohort. Moreover, strong physical interaction between AURKA and NUMB in their phosphorylated forms in the adjacent normal tissue, as evident from our experiments, was in accord with a previous study regarding involvement of G-protein coupled receptor/protein kinase C in the adjacent normal tissues to mediate NUMB phosphorylation by AURKA [54]. CSC markers have been shown to be enriched in the adjacent normal tissues in triple-negative breast cancer [55], and the mentioned cells have been reported to possess efficient DNA repair capability, thereby withstanding chemotherapy and radiation. Although in a different tissue model, in colon cancer, tumor-adjacent ‘normal’ tissues have been compared with healthy tissues, which indicated similarity of both the populations in terms of morphology, clonogenic abilities, and differentiation, except for the fact that tumor-adjacency ‘normal’ tissue is changed at the molecular level [56], resulting in EMT enrichment. Additionally, the importance of tumor adjacency in affecting disease prognosis as well as chances of relapse had been documented previously [43].

Our result, furthermore, showed an elevated expression of a crucial mesenchymal marker, vimentin, in the AURKA+ve cells of breast cancer tissue. Vimentin-expressing cells in blood, consequently, also demonstrated a positive correlation with Oct4/Sox2-expressing cells, which themselves did not show AURKA expression. This result, together with other published data stating the role of vimentin in tumor development and disease relapse due to persistence of drug-evading stem-like populations [57] guided us to look for the ABCG2 status in these Oct4/Sox2/vimentin+ve cells. The result clearly demonstrating ABCG2 positivity provided a clue that they represent a drug-escaping disseminated circulating cell population. When ABCG2 status was checked in patient tissues, two distinct subpopulations (ABCG2^high^ and ABCG2^low^) were noted. ABCG2^low^ subpopulation was increased in tumor along with up-regulated AURKA. This population further showed CD44/CD24 double positivity, indicating the hybrid stem-like population that had been reported previously to negatively affect prognosis [47]. ABCG2^high^ population was more abundant in adjacent normal and was mostly vimentin^high^AURKA^low^ and yet again CD44/CD24 double positive. This was in alliance with a previously published report related to vimentin overexpression with attainment of hybrid EMT states in breast cancer [58]. Therefore, it can be inferred that the disseminated cells available in patient blood might be both from the tumor and the adjacent normal parts, and most of the cells were identifiable and targetable using vimentin, including the quiescent ones lacking AURKA expression. However, considering the fact that AURKA-expressing cells in tumors retained stem-like molecular characteristics, as evident from their CD44/CD24 profile, AURKA targeting has to be taken into consideration for addressing these tumor residents.

The quiescent mesenchymal progeny lacking AURKA abundance will be efficiently targeted neither by chemotherapy nor by specific AURKA inhibitors because these agents are mostly designed to target cells undergoing an active cell cycle. Nevertheless, vimentin expression in these cells appears to be an ‘achilles heel’ that brings the promise of efficient addressal of these cells. The present findings are therefore encouraging enough to propose vimentin as a notable target, given the fact that vimentin is expressed even during the state of apparent AURKA negativity in blood. Vimentin targeting was reported to reduce the toxicity significantly by selecting only the mesenchymal or the epithelial cells undergoing EMT [59]. Vimentin targeting compounds could also provide an added advantage by reducing infection vulnerability [60] since extracellular vimentin is used as an attachment factor for a range of pathogens. However, an effective detection system for CSCs utilizing vimentin has yet to have its importance in routine clinical practice.

Recent research advocated the repurposing of statins as agents directed against vimentin [61]. Although specific vimentin inhibitors are available, statin repurposing seemed feasible clinically, owing to their acclaimed tolerability in patients [62] and beneficial bystander effects like betterment of endothelial activity, lowered chances of Alzheimer’s, dementia, ischemic stroke, and anti-inflammatory effects [63]. By building on established safety and pharmacokinetics data, drug repurposing reduces the time and cost to expand therapeutic options in oncology by acting as an alternative to de novo discovery of drugs, which would require more extensive trials before coming into clinical use. Available drugs with established safety and pharmacokinetic data provide the advantage of repurposing owing to the curtailment of time and cost but add the benefit of expanding feasible therapeutic options in oncology by acting as an alternative to *de novo* discovery of drugs [64]. Statins particularly the lipophilic ones like atorvastatin may be considered as effective candidates for vimentin targeting due to their reliance on vimentin positivity for better efficacy [65] and have also been associated with a reduced chance of developing breast cancer [66]. Atorvastatin is also reported to reduce vimentin expression [38]. Our results, which showed reduced vimentin expression in mammospheres upon atorvastatin administration, also aligned with the earlier reports. This may explain our findings where atorvastatin efficiently counteracted migration and mammosphere formation in highly vimentin-expressing MDA-MB-231. It is worth mentioning that there are instances of Oct4+vimentin+cells being positively correlated with tumor grade, lymph node metastasis, infiltration of surrounding tissues, and vasculogenic mimicry in gallbladder adenocarcinoma [67]. On the other hand, isoforms of Oct4 have been identified [68] in bone-marrow-derived mesenchymal stem cells (BM MSC), which are recruited to tumor microenvironment for effective immune suppression as reported in colorectal cancer [69]. Importantly, BM MSCs may also be mobilized to blood [70] in cancer patients. Therefore, it is necessary to analyze the complete profile of the Oct4+ve cells from patient blood before isolation and *ex vivo* co-culture of the same for studying possible pro-tumorigenic activities. Another area of further study involves probable extracellular transport of Sox2 or its targets, which happen to participate in AURKA transcriptional up-regulation with Oct4 even in the absence of DNA binding. Extracellular transportation of Oct4 [71] has already been documented; however, there is to date, no such data regarding Sox2. The mentioned studies will be able to explain the underlying reason for obtaining a positive correlation of Oct4/Sox2-expressing cells with AURKA-expressing cells without having a similar correlation in MFI in blood. Elaborate analysis in this context is needed to obtain the underlying scenario of CSC plasticity from tumor initiation to distant metastasis. This study, although restricted by its low cohort size, still brings into light a peripheral blood-based approach, including vimentin among other markers for the detection of BCSC populations, thus forming the basis for future studies in a larger population cohort. This study may catalyze the development of a simple flow cytometry-based approach for the detection of BCSCs in routine clinical practices with further extensive research and thus brings the promise of a combination therapy including AURKA inhibitor alisertib and atorvastatin for efficient BCSC addressal.

## Supplementary Material

Supplementary Figures S1-S8 and Tables S1-S2

## Data Availability

Publicly available datasets with accession numbers GSE206724 was analyzed in this study. These data can be found at https://www.ncbi.nlm.nih.gov/gds/. Transcript expression in tumour versus normal samples from GEPIA2 platform [72] available at https://gepia2.cancer-pku.cn/. Kaplan–Meier plotter with selected dataset 208079_s_at https://kmplot.com/analysis/index.php?p=service&cancer=breast was used for survival analysis. ALGGEN-PROMO webtool was used for assessing the transcription factor binding sites available at http://www.lsi.upc.es/∼alggen. Correlation analysis between vimentin and top 50 mesenchymal genes was performed using the microarray data from Alsuliman et. al EMT signature genes 2015–17 (Breast cancer, PMID 26245467) as available in EMTome available at http://www.emtome.org. Association of vimentin with stemness was assessed in StemChecker available at http://stemchecker.sysbiolab.eu. Druggability assessment was perfomed in SPIDER available at http://pmlabstack.pythonanywhere.com/SPIDER. Drug repurposing potential was checked in RepurposeDrugs available at https://repurposedrugs.org/. All other data are available from corresponding author on reasonable request.

## Abbreviations

AURKA: Aurora Kinase A
BC: Breast Cancer
BCSCs: Breast Cancer Stem Cells
BM MSC: Bone-Marrow-derived Mesenchymal Stem Cells
ChIP: Chromatin Immunoprecipitation
Co-IP: Co-Immunoprecipitation
CSCs: Cancer Stem Cells
DEGs: Differentially Expressed Genes
DMSO: Dimethyl Sulphoxide
EMT: Epithelial-Mesenchymal Transition
FDR: False Discovery Rate
GEPIA2: Gene Expression Profiling Interactive Analysis
GLOBOCAN: Global Cancer Observatory
GTeX: the Genotype-Tissue Expression
KEGG: Kyoto Encyclopedia of Genes and Genomes
MFIs: Mean Fluorescent Intensities
Oct4: Octamer-binding Transcription Factor 4
PBS: Phosphate Buffered Saline
RFS: Relapse-free Survival
Sox2: Sex-determining Region Y-box 2
TCGA: The Cancer Genome Atlas
