# Supplementary material for: A peripheral blood-based approach involving vimentin along with AURKA enabled efficient tracking of elusive Oct4/Sox2-expressing disseminated breast cancer stem cells
Source: Biosci Rep. 2026 Jun 24;46(7):BSR20253828. doi: 10.1042/BSR20253828 (PMC13305960; doi:10.1042/BSR20253828)
Supplement: Supplementary Figures S1-S8 and Tables S1-S2 [file BSR-2025-3828_supp.pdf]

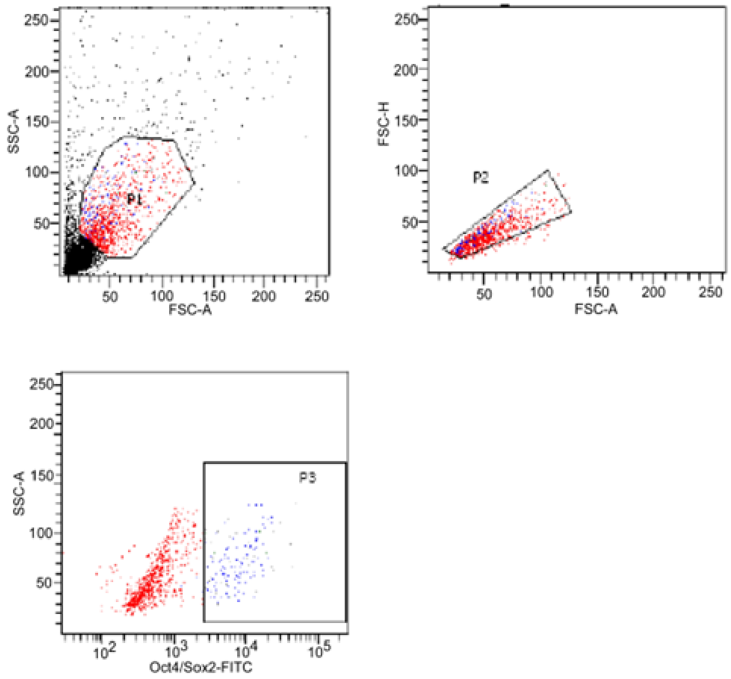

Supplementary figure 1: Gating strategy for identification of Oct4/Sox2 positive cells in patient tissues or cell lines

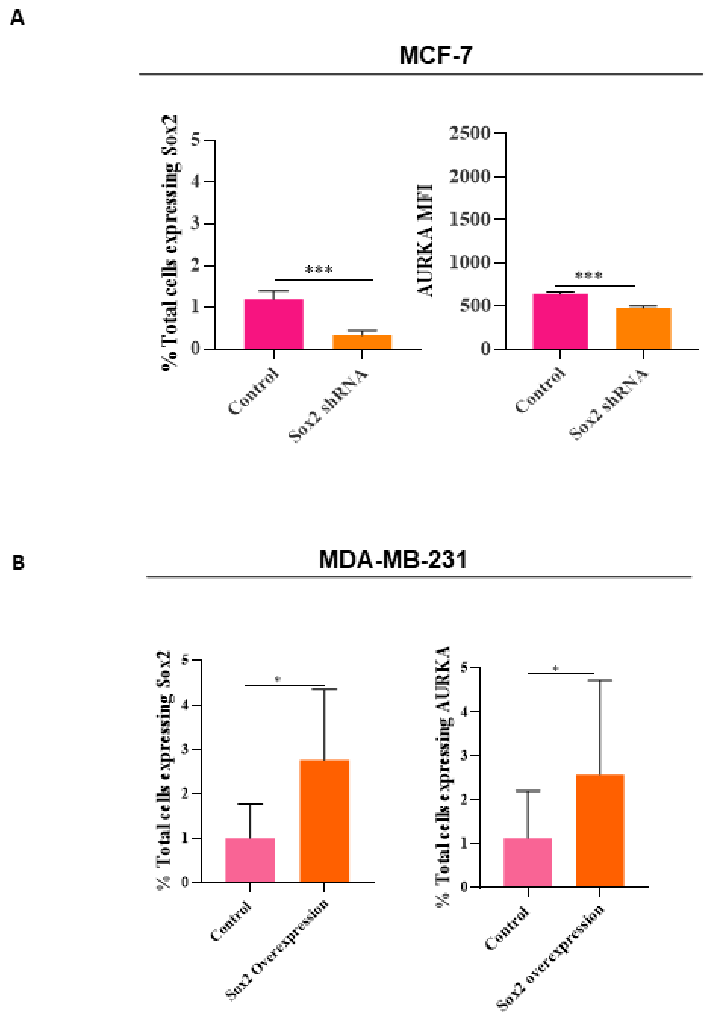

Supplementary figure 2 : (A). Interleaved bar diagrams representing downregulation of AURKA under silencing of Sox2 in MCF-7 as observed by flow cytometric analysis. Left panels represent efficiency of transfection in terms of percentage of positive cells and the right panels represent mean fluorescent intensities (MFIs) of AURKA positive cells under vector control vs. shRNA transfection. **(B). I**ncreased percentage of Sox2 positive cells upon overexpression (Left Panel) resulted into augmented AURKA positive cell population (%) as evident from the Right Panel.

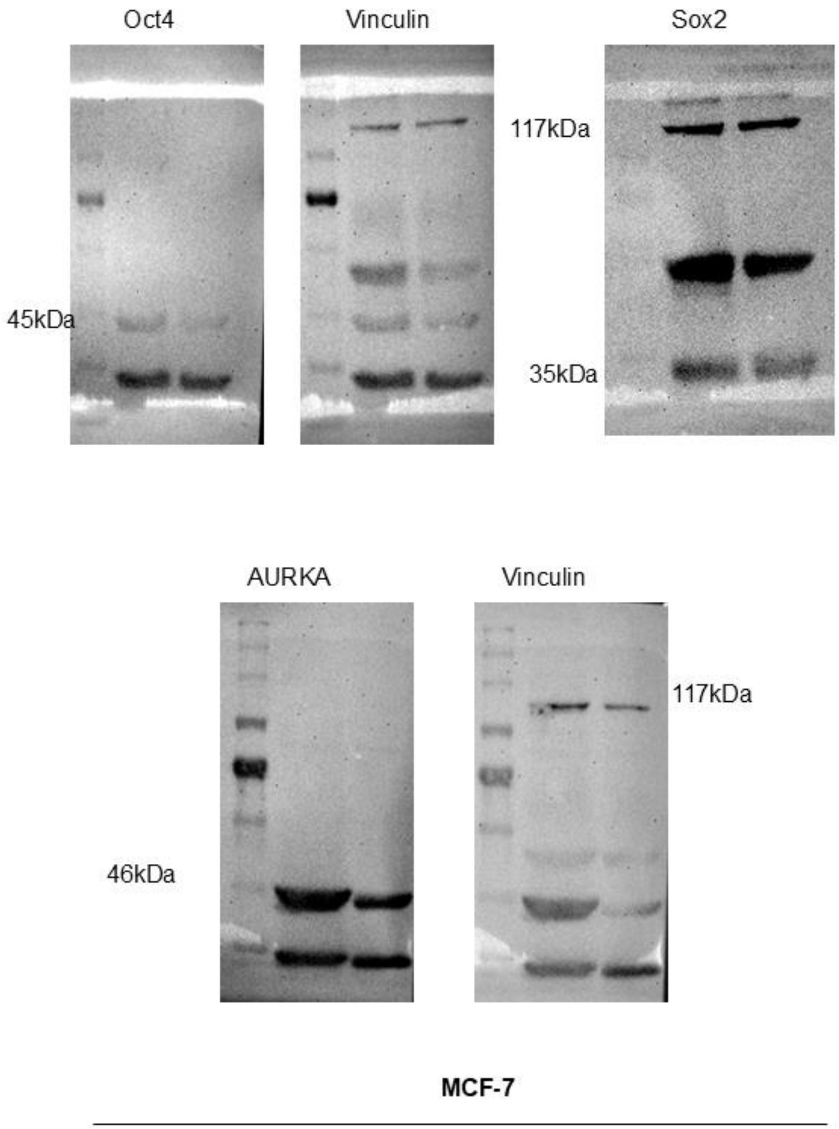

Supplementary figure 3 : Total raw blot images of Oct4, Sox2, AURKA and Vinculin as obtained from Western Blot Results in MCF-7 cells upon knockdown of Oct4

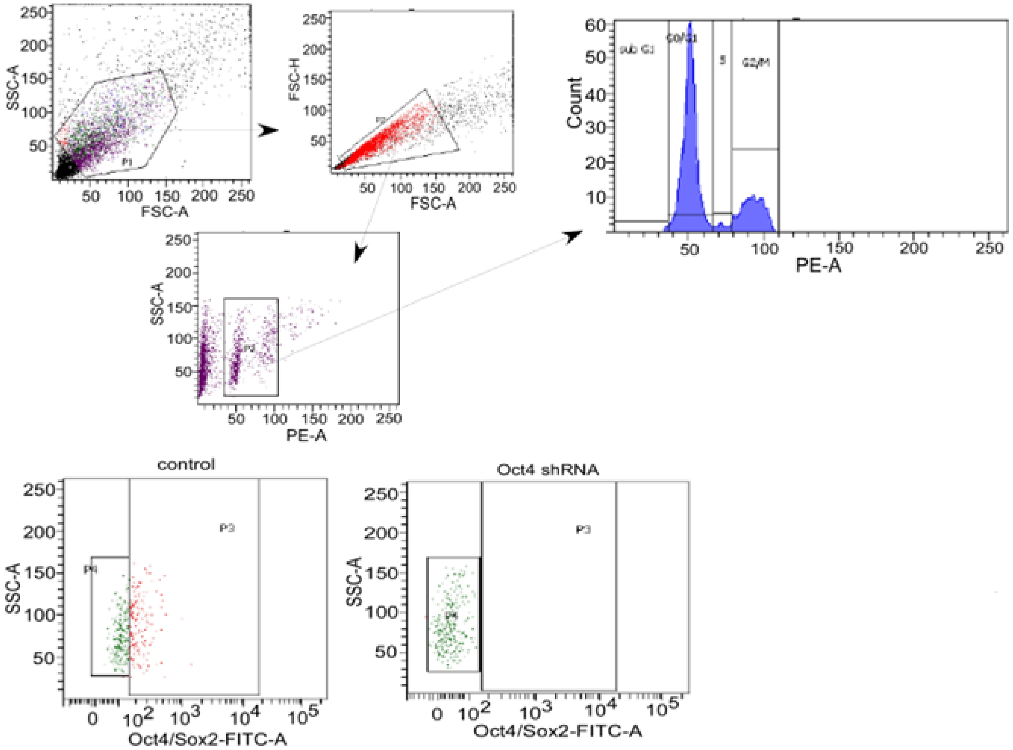

Supplementary figure 4: Gating strategy for cell cycle analysis **(Upper Row)** and Gating strategy for checking shRNA transfection efficiency **(Lower Row)**

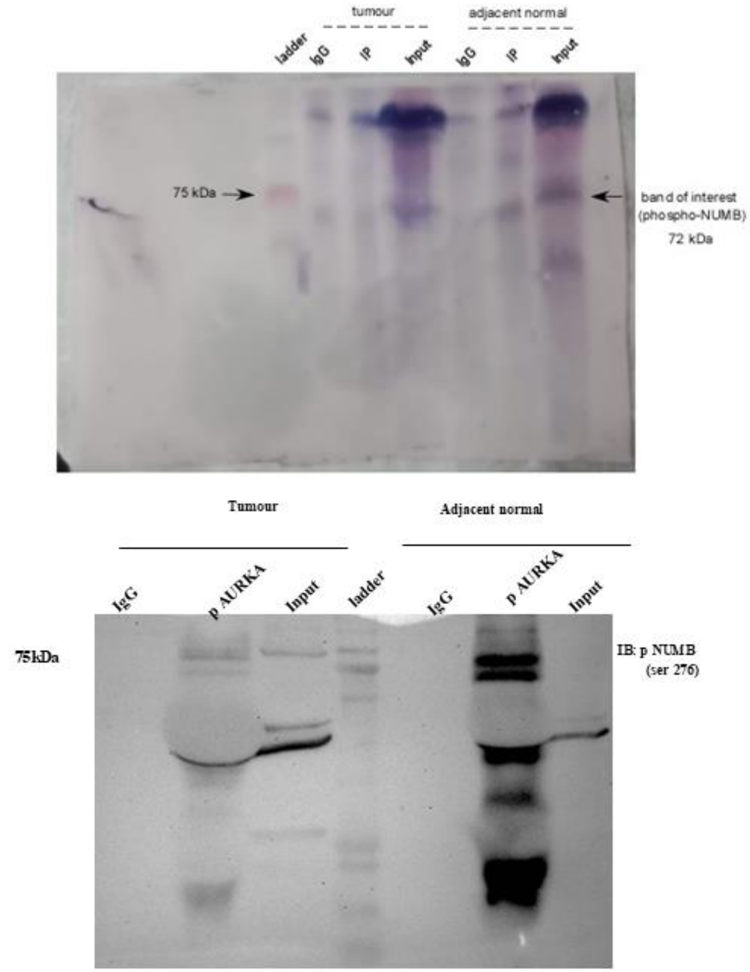

Supplementary figure 5 : Full Blot for Co-IP with phospho AURKA(T288) and IB with phospho NUMB (S276) in two different breast cancer tissue samples

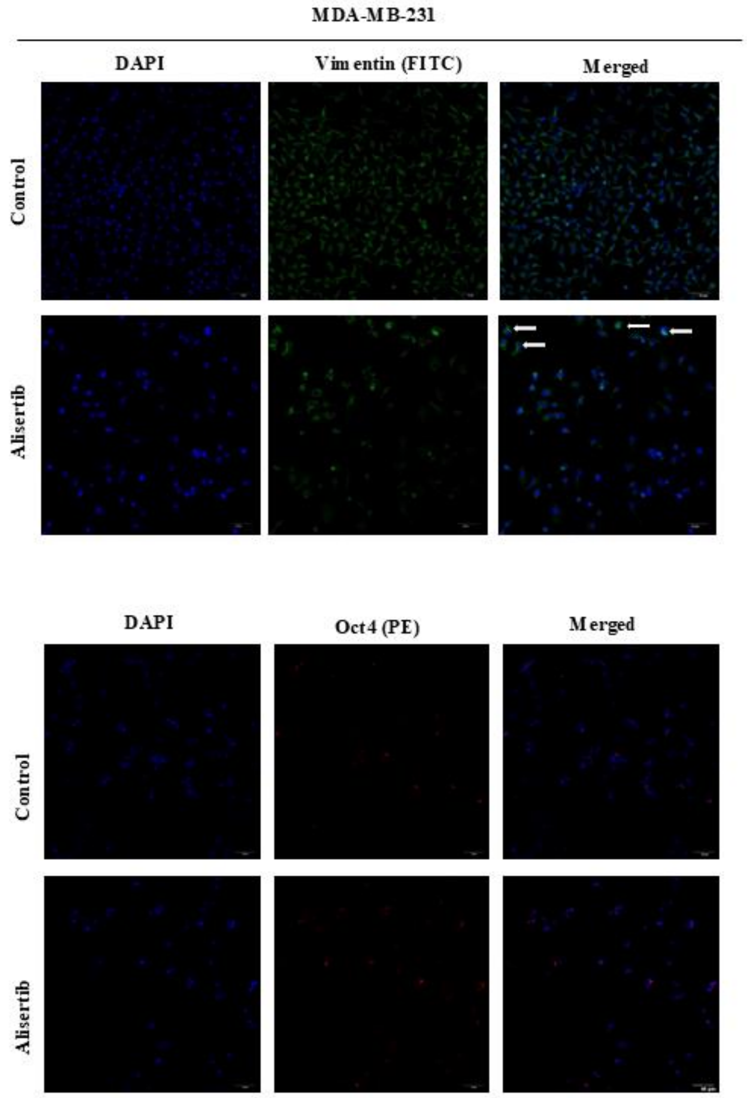

Supplementary figure 6: Confocal microscopic images A. vimentin expression in untreated or alisertib treated MDA-MB-231. B. Oct4 expression in untreated or alisertib treated MDA-MB-231. Magnification 20x, Scale Bar: 60 μM. White arrows indicate cells with maintained vimentin expression even after alisertib treatment.

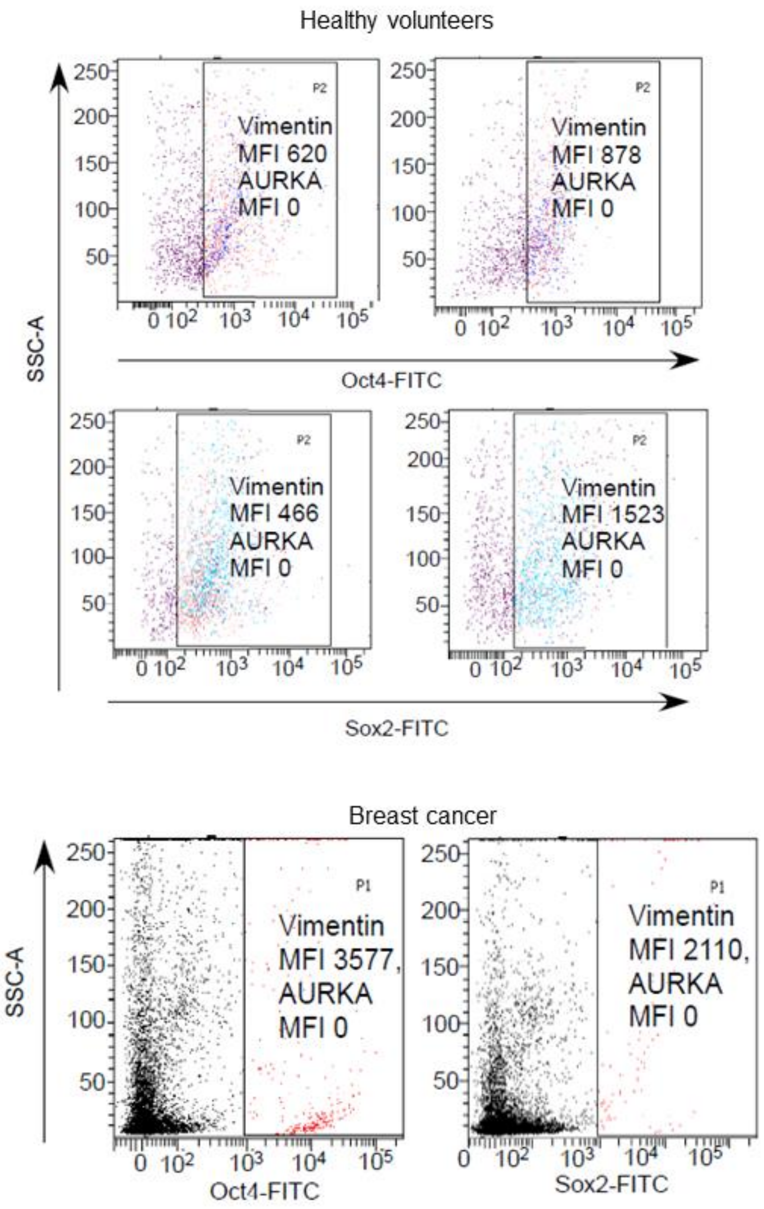

Supplementary figure 7: Representative flow cytometric dot plot showing differential vimentin expression in terms of MFI in healthy vs breast cancer patient

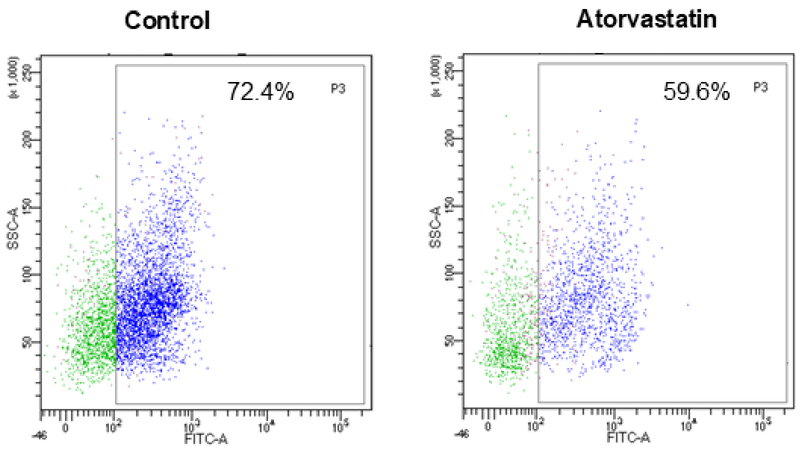

Supplementary figure 8: Representative flow cytometric dot plot showing percentage of vimentin expressing mammospheres under untreated or Atorvastatin treated condition.

Supplementary Table 1: Calculated Correlation Values between Oct4/Sox2/AURKA of Tumor and Adjacent Normal Cases (n=15)

**Correlation coefficient (r Value)**	Oct4 AN	Oct4 T	Sox2 AN	Sox2 T	AURKA (O) AN	AURKA (O) T	AURKA (S) AN	AURKA (S) T
Oct4 AN	1	**0.981360509**	**0.932556313**	**0.95622061**	**0.545474613**	**0.586525477**	0.086057486	**0.633584803**
Oct4 T	**0.981360509**	1	**0.855477619**	**0.94872051**	0.446201877	0.441731794	-0.029300255	**0.563099017**
Sox2 AN	**0.932556313**	**0.855477619**	1	**0.863781**	**0.747416982**	**0.820319947**	0.37044649	**0.794813497**
Sox2 T	**0.956220605**	**0.948720507**	**0.863780995**	1	0.349410599	**0.535938653**	-0.075225293	**0.646478441**
AURKA (O) AN	**0.545474613**	0.446201877	**0.747416982**	0.3494106	1	**0.778570501**	**0.761317194**	**0.676290599**
AURKA (O) T	**0.586525477**	0.441731794	**0.820319947**	**0.53593865**	**0.778570501**	1	**0.635868179**	**0.814679917**
AURKA (S) AN		-0.029300255	0.37044649	-0.0752253	**0.761317194**	**0.635868179**	1	0.50967161
AURKA (S) T	**0.633584803**	**0.563099017**	**0.794813497**	**0.64647844**	**0.676290599**	**0.814679917**	0.50967161	1

Supplementary Table 2: Calculated Significance Value using GraphPad-Prism software (8.0.1).

**p Value**	Oct4 AN	Oct4 T	Sox2 AN	Sox2 T	AURKA (O) AN	AURKA (O) T	AURKA (S) AN	AURKA (S) T
Oct4 AN	0	1.07591E-10	4.08016E-07	2.60531E-08	0.03545102	0.021550392	0.760404236	0.011213595
Oct4 T	1.07591E-10	0	4.77373E-05	7.15036E-08	0.095481752	0.0992532	0.917442562	0.028841408
Sox2 AN	4.08016E-07	4.77373E-05	0	3.31836E-05	0.001360085	0.000179744	0.17407755	0.000398848
Sox2 T	2.60531E-08	7.15036E-08	3.31836E-05	0	0.201757058	0.039468537	0.78989448	0.009207375
AURKA (O) AN	0.03545102	0.095481752	0.001360085	0.201757058	0	0.000627366	0.000976556	0.005636156
AURKA (O) T	0.021550392	0.0992532	0.000179744	0.039468537	0.000627366	0	0.010835586	0.000216576
AURKA (S) AN	0.760404236	0.917442562	0.17407755	0.78989448	0.000976556	0.010835586	0	0.052285945
AURKA (S) T	0.011213595	0.028841408	0.000398848	0.009207375	0.005636156	0.000216576	0.052285945	0
